# Safe Stockpiling of the MTX‑1 Primary Explosive in Alkali or Alkaline Earth Metal Complexes and Coordination Polymers

**DOI:** 10.1021/acs.inorgchem.5c04892

**Published:** 2026-02-18

**Authors:** Maksim A. Samsonov, Jakub Mikuláštík, Robert Matyáš, Aleš Růžička

**Affiliations:** † Department of General and Inorganic Chemistry, Faculty of Chemical Technology, 48252 University of Pardubice, Studentská 573, Pardubice 532 10, Czech Republic; ∥ Institute of Energetic Materials, Faculty of Chemical Technology, 48252 University of Pardubice, Studentská 573, Pardubice 532 10, Czech Republic

## Abstract

The safe stockpiling of high-energy combustible and explosive materials is important for environmental and human population protection, particularly in mining and military areas. **MTX-1** (2-(tetrazol-5-yl-diazenyl)­guanidine) is used in percussion primer compositions that react rapidly with the hydroxides of alkali or alkaline earth metals to form complexes of diverse composition. These are insensitive to mechanical stimuli and to intense heat under high confinement. The most stable complexes were found to be those with ions having the smallest effective ion radii (Mg^2^^+^ and Ca^2^^+^), followed by Li^+^. These complexes form discrete mononuclear (Mg^2^^+^ and Ca^2^^+^) or dimeric (Li^+^) structures in the solid state. One-dimensional (1D) (Na^+^, Sr^2^^+^, and Ba^2^^+^), 2D (K^+^ and Rb^+^), and 3D (Cs^+^) coordination polymeric structures were found for the remaining complexes with larger ions. Detailed topological analysis of the electron density and quantitative estimation of π–π stacking interactions were performed. Complexes could be used for intense flame coloring in pyrotechnics. Upon thermal treatment of complexes over ∼200 °C, water is lost from the coordination sphere of the metal ion, which leads to materials that are slightly sensitive and rehydrate in moist air. Recovery of insoluble **MTX-1** is possible when these complexes are treated with an aqueous acid.

## Graphical abstract

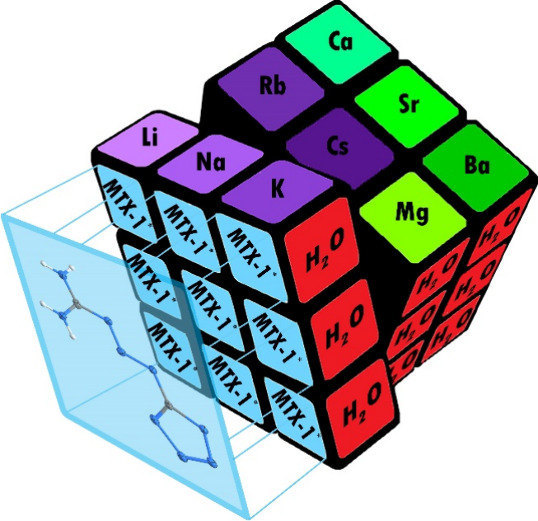

## Introduction

A critical challenge in the field of energetic materials science is ensuring safe storage and handling of primary explosives. A modern approach to the development of energetic materials involves the creation of Energetic Coordination Polymers (ECPs),^1^ where active components are stored in crystalline form as metal–organic complexes. The preparation of such metal complexes is limited by several difficulties during synthesis, such as the stability of compounds in media, the use of organic solvents, and higher temperatures. These compounds belong to the metal–organic framework (MOF) materials family. Unlike MOFs with stable ligands, they are relatively unexplored but have great potential and can offer several advantages over traditional organic explosives. A crucial role in their behavior is played by intermolecular interactions, which influence the stability of the material and its detonation properties.^2–4^

The performance of primary explosives usually correlates with a highly positive heat of formation. However, the number of nitrogen atoms within a molecule can also be used as an auxiliary criterion for its prediction. The substituted tetrazole group was identified as the perfect candidate for the formation of stable anions and, consequently, coordination compounds. Furthermore, the tetrazole group exhibits unique properties, leading to its compounds being utilized in a broad spectrum of applications across various fields of chemistry.^5^ Given that the tetrazole group exhibits biological activity, these compounds have therefore been widely used in biochemistry and pharmacology in recent decades.^6–10^ The use of tetrazoles has also been mentioned in other areas of chemistry, such as photochemistry^11^ and coordination chemistry,^12,13^ as well as in the production of nanostructured materials.^14^ Substituted tetrazoles with a high nitrogen content are of particular interest due to their unique properties as energetic materials. Many of these compounds have been proposed as primary or secondary explosives or valuable components in modern propellants and pyrotechnic compositions,^15,16^ and some are used in the explosives industry.^17,18^ Over the past two decades, numerous lithium, sodium, strontium and barium salts of various tetrazole derivatives have been reported as effective colorants for modern pyrotechnics.^19–23^

The oldest tetrazole still in extensive use today is 5-[(1E)-3-amidiniotetraz-1-en-1-yl]­tetrazolide hydrate, commonly called tetrazene (Figure 1). This compound has been used for almost a century as a key energetic sensitizer, particularly in priming and stabilizing compositions. However, this substance has one major drawback: low thermal and hydrolytic stability, which affects the stability of the resulting composition.^24–26^

**Figure 1:**
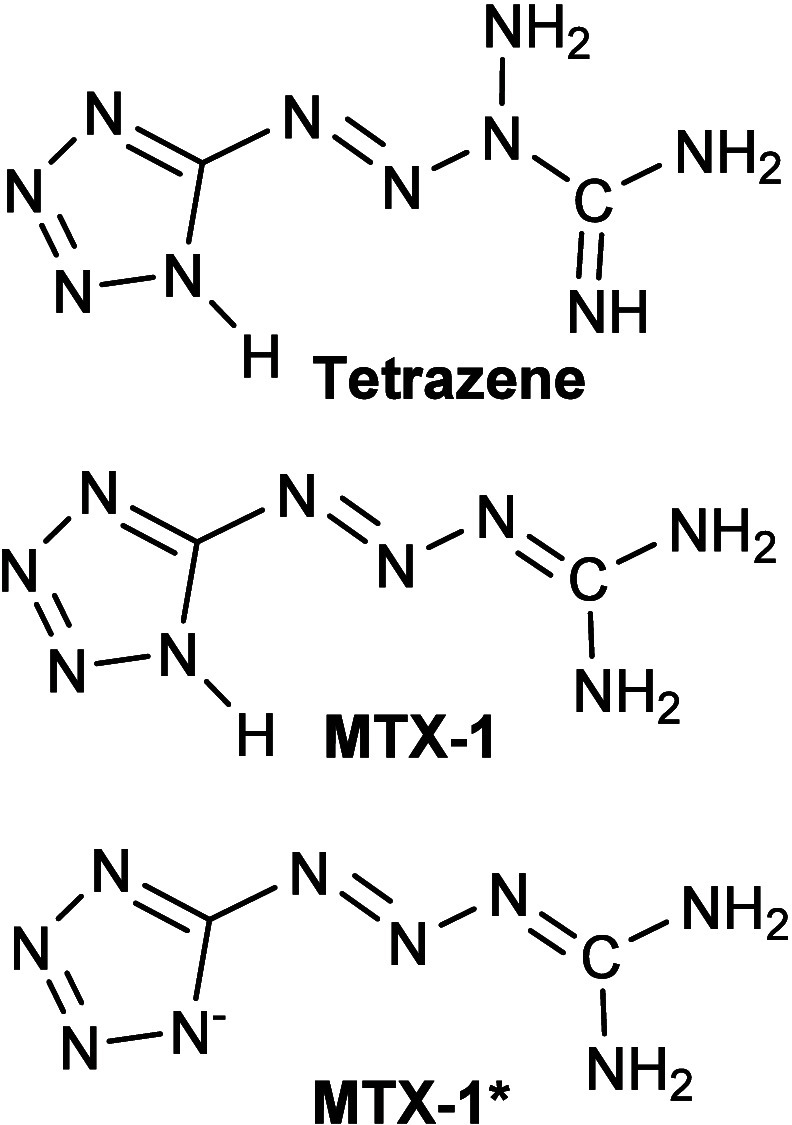
Canonical formulas of tetrazene, **MTX-1**, and its deprotonated form MTX-1*.

In 2010, Fronabarger and Williams introduced an alternative to tetrazene in the form of a structurally similar compound 2-(tetrazol-5-yl-diazenyl)­guanidine (**MTX-1**; see Figure 1). This compound exhibits significantly higher hydrolytic stability and has been suggested as a chemically stable alternative to tetrazene for use in primer compositions.^27,28^

Unlike tetrazene, which decomposes under alkaline conditions, **MTX-1** can be easily deprotonated to form anions (**MTX-1** *). It also forms salts and complexes with various bases. X-ray structure determination has only been performed for the cesium salt thus far.^27,28^ High nitrogen compounds such as **MTX-1** have a high positive heat of formation (383 ^28^ or 314 kJ/mol^29^), resulting in higher combustion temperatures—a key factor in achieving an intense, bright flame color. The low carbon content of the molecule also limits the formation of soot, which washes out the desired flame color by producing undesirable blackbody radiation. These properties can enhance the efficiency of color generation, enabling reduced concentrations of colorants while improving flame purity compared to traditional compositions.^30–32^

This study focuses on the synthesis, properties, and structural characterization of a comprehensive series of **MTX-1** complexes of alkali and alkaline earth metals. Particular interest is devoted to using these compounds as colorants for modern color flame compositions. The correlation between metal type, crystal structure, supramolecular architecture, intermolecular forces, and the properties of these pyrotechnic materials is investigated in order to overcome obvious problems with the transportation and storage of **MTX-1**.

## Results and Discussion

The general synthetic route exploits the acidic nature of the tetrazole ring to facilitate a simple acid–base reaction (Figure 2). This involves reacting **MTX-1** with the corresponding metal hydroxide in an aqueous or aqueous-methanolic solution. The initially insoluble **MTX-1** readily dissolves upon deprotonation, forming clear yellow-to-orange solutions of the respective salt. All of the salts obtained formed hydrates (or methanolates), which were crucially found to be stable under ambient laboratory conditions, showing no signs of decomposition or a spontaneous loss of water over time.

**Figure 2:**
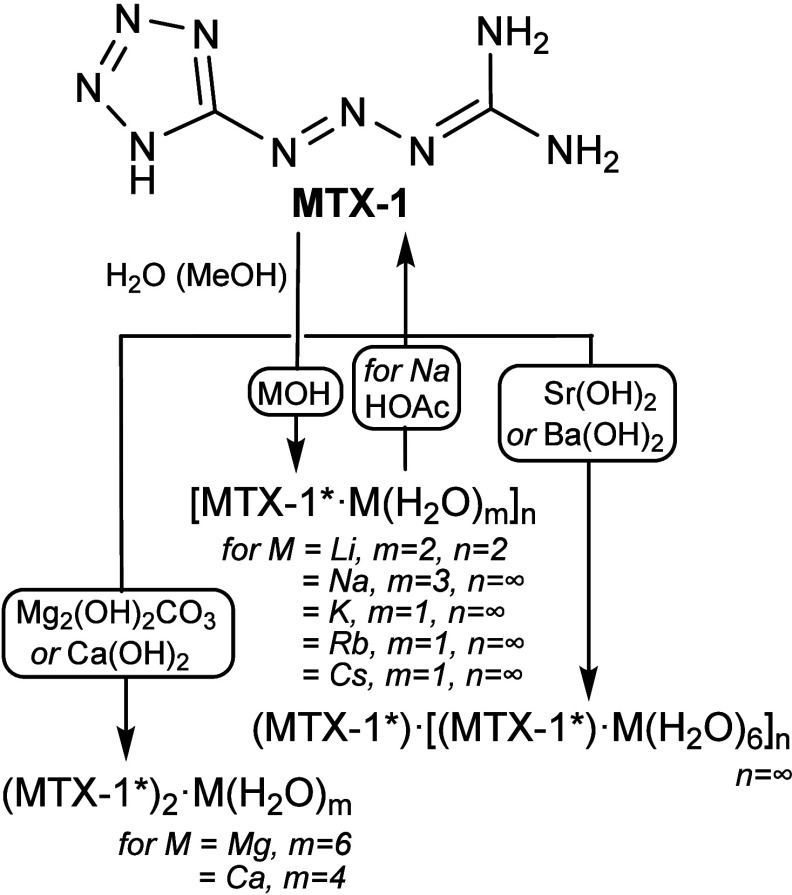
Preparation of MTX-1* complexes.

The resulting alkali metal salts generally exhibited high solubility in water and methanol, moderate solubility in ethanol, and complete insolubility in acetone. This property is used during recrystallization to produce a good yield through antisolvent precipitation. Attempting to prepare anhydrous single crystals of sodium salt from hot, dry methanol produced an unexpected outcome. Rather than the desired anhydrous product, a stable methanol solvate, **[MTX-1** * **·Na­(MeOH)**_**2**_ **]**, crystallized upon cooling. IR spectroscopy also suggested a similar tendency to form a methanol adduct for the lithium salt.

The key objective of this study was to evaluate the effect of salt formation on the energetic properties of **MTX-1**, with the aim of reducing its sensitivity to enable safer handling. As expected, all of the prepared hydrated complexes exhibited excellent insensitivity to mechanical stimuli (impact *E*_5__0_ > 50 J and friction *F*_5__0_ > 360 N), which demonstrated the profound desensitizing effect of coordinated water.

In order to reveal the intrinsic energetic characteristics of the **MTX-1** * anion in the presence of different cations, dehydration of the complexes was necessary. However, this process revealed the critical role of water in stabilizing the structure, as the Li, Mg, and Ca complexes could not be dehydrated without decomposing. Of the salts that were successfully dehydrated (Na, K, Rb, Cs, Sr and Ba), a clear trend emerged in the alkali metal series: sensitivity to impact and friction increased with ionic radius (Na^+^ < K^+^ < Rb^+^ < Cs^+^) and influence of the crystal packing and an ability to coordinate water molecules. However, all salts remained significantly less sensitive than parent **MTX-1** (see Table 1 and Figure 3). This trend is consistent with reports on other energetic salts^33,34^ and can be attributed to the decreasing charge density of the cation. In contrast, no clear trend was discernible for Sr and Ba, despite their similar sensitivity. Initially, all salts exhibited only a mild response: a faint crackle and a puff of smoke. This is in sharp contrast to the violent deflagration response of pure **MTX-1**.

**Table 1:** Impact Energy (*E*_50_) and Friction Force (*F*_50_) of 50% Probability to Initiation and Decomposition Temperatures (*T*_dec_) of **MTX-1** and Its Dehydrated and Non-Dehydrated Metal Salts and Effective Ionic Radii (*r*_eff_)^39^ of Relative Cations

compound	impact sensitivity *E*_50_ [J]	friction sensitivity *F*_50_ [N]	temperature of decomposition (DTA, onset) *T*_dec_ [°C]	effective ionic radii *r*_eff_, [pm], (*r*_eff_/charge)
**MTX-1**	1.2	5.5	195	–
Li	>50	>360	229	73 (73)
Na	5.5	353	248	116 (116)
K	2.7	345	241	169 (169)
Rb	2.5	278	235	180 (180)
Cs	1.8	190	224	195 (195)
Mg	>50	>360	200	86 (43)
Ca	>50	>360	232	114 (57)
Sr	3.1	362	230	135 (67.5)
Ba	5.8	290	234	152 (76)

**Figure 3:**
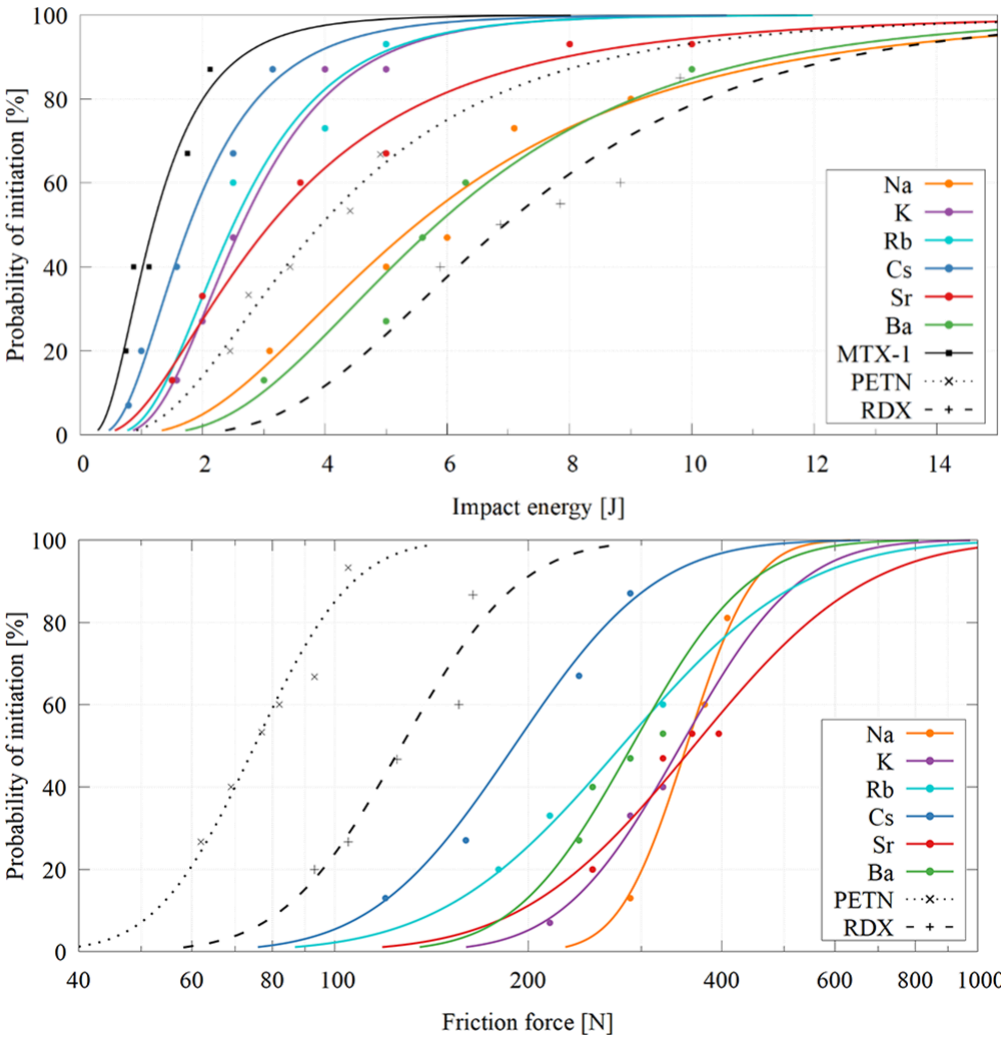
Impact sensitivity (top) and friction sensitivity (bottom) curves for **MTX-1** and its dehydrated salts compared with standard explosives PETN and RDX.

The detailed results of the sensitivity tests are included in the Supporting Information (Tables S2 and S3). In addition to their reduced sensitivity, the salts exhibit improved thermal stability, with decomposition temperatures (*T*_dec_ ≥ 224 °C) that are consistently higher than those of **MTX-1** (195 °C). This difference is attributed to charge delocalization across the **MTX-1** * anion. A trend of decreasing thermal stability with an increasing cation size was also observed for the anhydrous alkali salts. This suggests that while the charge delocalization ensures the higher thermal stability of the salts, the specific sensitivity limits are dominated by lattice properties, which become less favorable with increasing ionic radius, as has been discussed in the literature.^35–38^

The combination of high nitrogen content, reduced sensitivity, and the presence of specific metal cations (Li, Na, Sr, and Ba) makes several of these salts promising candidates for use as flame-coloring agents in pyrotechnic applications.

This is evident in the characteristic red flame produced by **[MTX-1** * **·Li­(H**_**2**_ **O)**_**2**_ **]**_**2**_ (Figure 4). From a handling and safety perspective, it is also noteworthy that the dehydrated sodium salt rehydrates to its original composition within one to two weeks under ambient conditions. The tendency to revert to the insensitive hydrated state underscores the fact that this is their most stable form, reinforcing its suitability as an inherently safe material.

**Figure 4:**
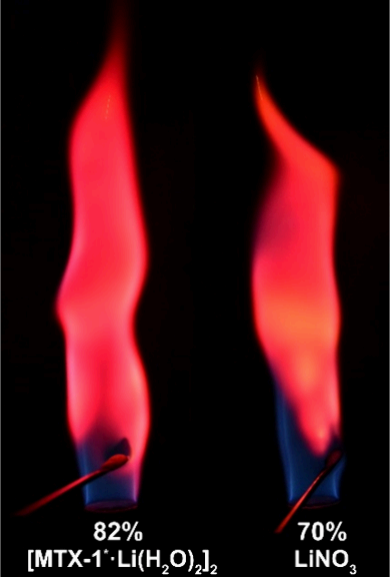
Flame coloration and color purity (%) produced by [MTX-1*·Li­(H_2_O)_2_]_2_ and LiNO_3_ in a gas burner flame.

### Na·MTX-1* as a Safe Storage Form of **MTX-1**

The ultimate goal of this strategy is to establish a complete and safe handling cycle for **MTX-1**. The sodium salt trihydrate **MTX-1** * **·Na­(H**_**2**_ **O)**_**3**_, serves as an excellent prototype for this concept. This cycle’s viability is demonstrated by the high efficiency of the key transitions: the salt is easily sequestered from the solution through energy-efficient antisolvent precipitation with acetone, and the parent **MTX-1** is nearly quantitatively regenerated through simple acidification (identity confirmed by FTIR and DTA, see Figure S1). Acetic acid is the ideal choice for this step because although other acids, such as nitric and methanesulfonic acids, are equally effective at precipitation, their nonvolatile nature complicates product purification. In contrast, any excess volatile acetic acid can be easily removed during drying. This establishes a practical, fully reversible pathway between the hazardous explosive and its safe carrier form in a quantitative process.

To further validate the safe stockpiling potential, the long-term and thermal stabilities of the sodium salt **[MTX-1** * **·Na­(H**_**2**_ **O)**_**3**_ **]**_***n***_ were investigated. Comparative analysis (FTIR, Raman, elemental analysis, and DTA, see Table S1 and Figure S12) of fresh samples and those stored for 6 months and 2 years under ambient conditions revealed no observable differences in physicochemical properties or signs of decomposition, confirming high long-term chemical stability over the investigated period. Furthermore, thermal stability was screened using a modified isothermal test at 75 °C for 48 h according to the *UN Manual of Tests and Criteria*, Test 3­(c).^40^ No ignition, explosion, or exothermic decomposition was observed during the test duration (stable baseline; no self-heating observed). While these initial results indicate promising stability, further comprehensive studies are required to fully qualify the material for industrial stockpiling.

Notably, the cornerstone of this cycle–the hydrated sodium salt–exhibits exceptional safety regarding external stimuli. It is insensitive to mechanical stimuli (impact >50 J; friction >360 N). Furthermore, it yielded a negative result in duplicate Koenen tests. The material underwent a prolonged, controlled burn of ∼90 s without rupture or deformation of the confining steel tube. Consequently, according to the UN *Recommendations on the Transport of Dangerous Goods “Orange Book”* regulations,^40^ this hydrate can be classified as insensitive to impact, and poses no explosion hazard under intense heat and high confinement. The intrinsic robustness conferred by salt formation was further investigated by subjecting the more sensitive anhydrous sodium salt to a detonation test. Remarkably, even this form failed to detonate from a 20 g Semtex 1A booster (Figures S13 and S14).

Taken together, these findings validate the salt formation strategy as a practical and complete handling cycle for **MTX-1**. This strategy sequesters hazardous explosives for safe storage and transport as its safe sodium salt trihydrate, which can then be quantitatively regenerated on demand.

According to X-ray structural analysis, the appropriate C–N and N–N separations are nearly identical for all compounds, with no difference between coordinated and noncoordinated **MTX-1** * anions, differing by no more than 0.03 Å (Figure 5). Crystallographic data for these complexes, along with other structural parameters, are summarized in Tables S3 and S4.

**Figure 5:**
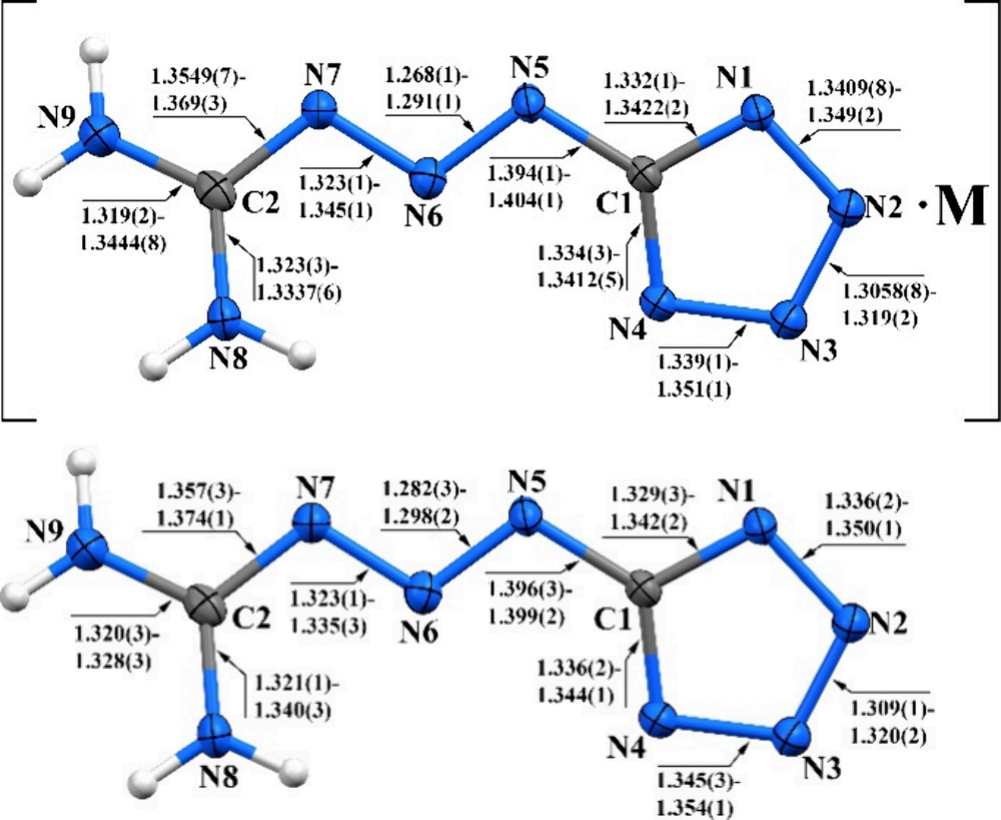
Bond length ranges for N–N and N–C bonds in coordinated (top) and noncoordinated (bottom) MTX-1* anions; M = Li, Na, K, Rb, Cs, Ca, Sr, Ba, and Mg.

### Mononuclear Complexes (MTX-1*)_2_·Mg­(H_2_O)_**6**_ and [(MTX-1*)_2_·Ca­(H_2_O)_4_] and the Dinuclear Complex [MTX-1*·Li­(H_2_O)_2_]_2_

The coordination polyhedron of the magnesium ion adopts a tetragonal bipyramidal geometry with six water molecules occupying the coordination sites. Notably, **MTX-1** * ligands are not coordinated to the metal center but instead form parallel head-to-tail π–π stacking arrangements in the crystal lattice, where the “head” corresponds to the tetrazole fragment (C1–N1–N2–N3–N4) and the “tail” to the guanidine moiety (N5–N6–N7–C2­(N8, N9) (Figure 6).

**Figure 6:**
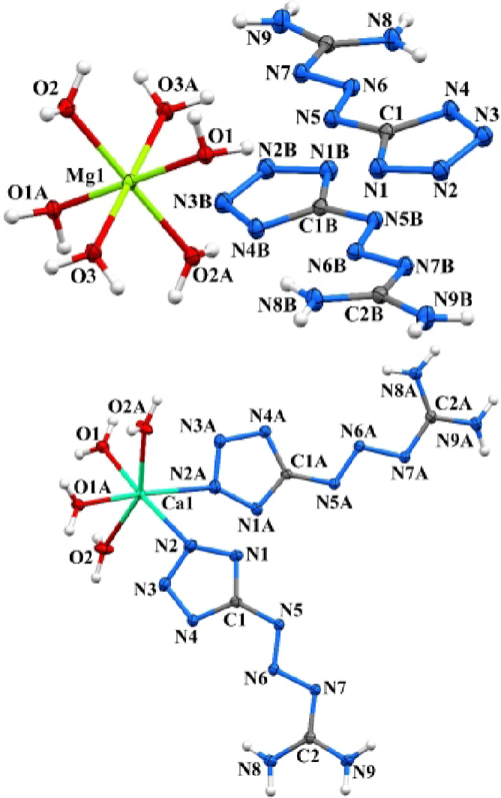
Molecular structures of (MTX-1*)_2_·Mg­(H_2_O)_6_ and [(MTX-1*)_2_·Ca­(H_2_O)_4_]. An ORTEP diagram, with a 50% probability level. Symmetry codes: (MTX-1*)_2_·Mg­(H_2_O)_6_: (A) 1–*x*, 1–*y*, −*z*; (B) 1–*x*, 1–*y*, 1–*z*; [(MTX-1*)_2_·Ca­(H_2_O)_4_]: (A) 1.5–*x*, 1/2–*y*, *z*.

In contrast, the calcium cation in **[(MTX-1** * **)**_**2**_ **·Ca­(H**_**2**_ **O)**_**4**_ **]** is coordinated by two **MTX-1** * anions and four water molecules, forming a slightly distorted tetragonal pyramidal geometry. The **MTX-1** * ligands are arranged in parallel head-to-tail π–π stacking, similar to the Mg complex, but an additional head-to-head stacking motif is also observed.

In the solid state, **[MTX-1** * **·Li­(H**_**2**_ **O)**_**2**_ **]**_**2**_ adopts a dimeric structure in which the lithium cation exhibits a tetrahedral coordination environment (Figure 7, Figure S16, and Scheme 1). Due to the cyclic nature of the structure, it can formally be classified as a 0D coordination polymer. The crystal packing of the Li complex consists of parallel stacks in which oppositely oriented **MTX-1** * ligands form π–π stacking interactions.

**Figure 7:**
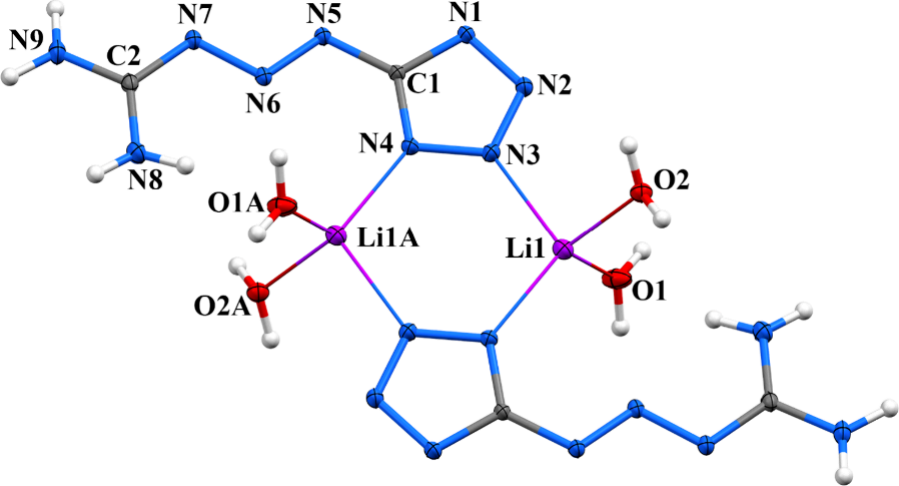
Molecular structure of [MTX-1*·Li­(H_2_O)_2_]_2_. An ORTEP diagram, with a 50% probability level. Symmetry codes: (A) −*x*, −*y*, and −*z*.

**Scheme 1:**
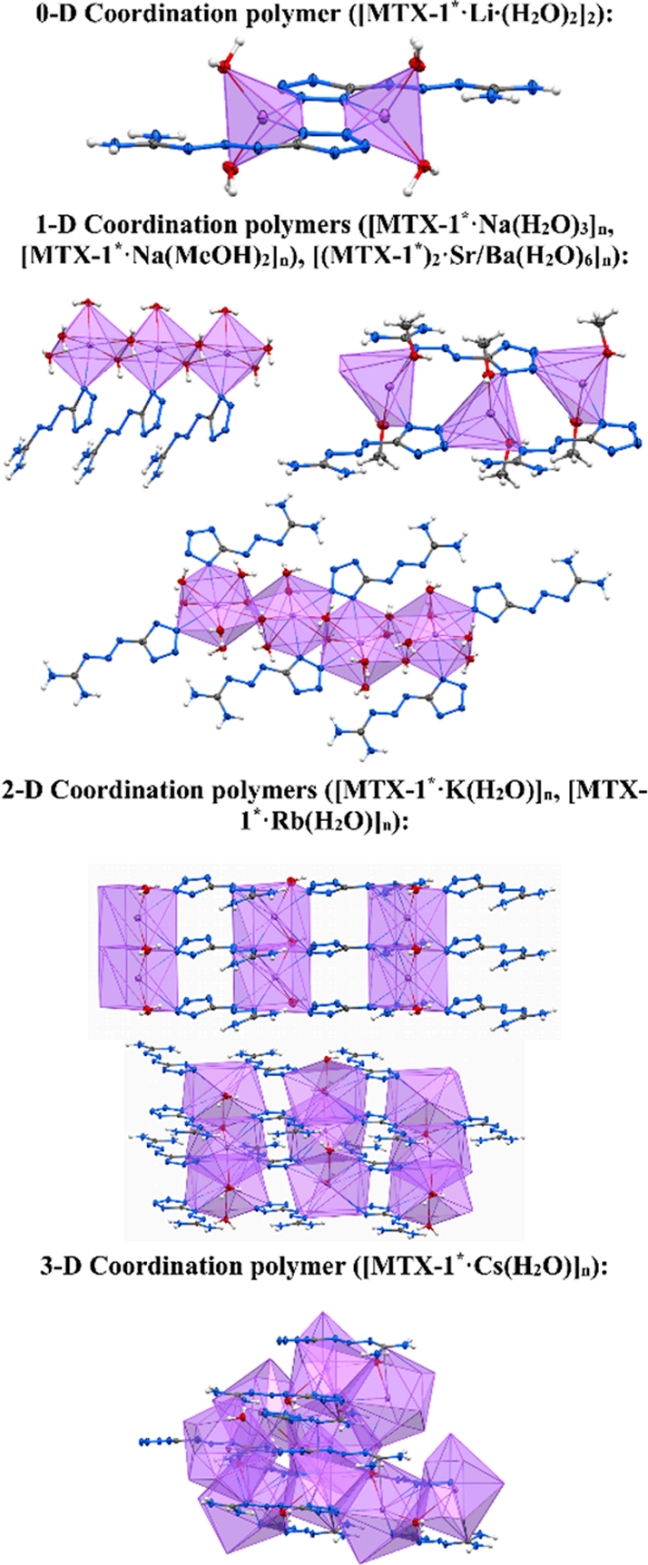
General View of Coordination Polymer Motifs for Li–Cs, Sr, and Ba Complexes of MTX-1*

### 1D Coordination Polymers: [MTX-1*·Na­(H_2_ **O)**_3_ **]**_*n*_, [MTX-1*·Na­(MeOH)_2_]_***n***_, (MTX-1*)·[MTX-1*·Sr­(H_2_O)_6_]_*n*_, and (MTX-1*)·[MTX-1*·Ba­(H_2_O)_6_]_*n*_

Complex **[MTX-1** * **·Na­(H**_**2**_ **O)**_**3**_ **]**_***n***_ forms 1D coordination polymer chains oriented along the *a*-axis (Figure 8, Figure S17, and Scheme 1). The monomeric units are connected by bridging water molecules. The coordination polyhedra of the sodium cation adopt a slightly distorted tetragonal bipyramidal geometry. In the crystal packing, **MTX-1** * molecules are arranged parallel to each other with a small shift.

**Figure 8:**
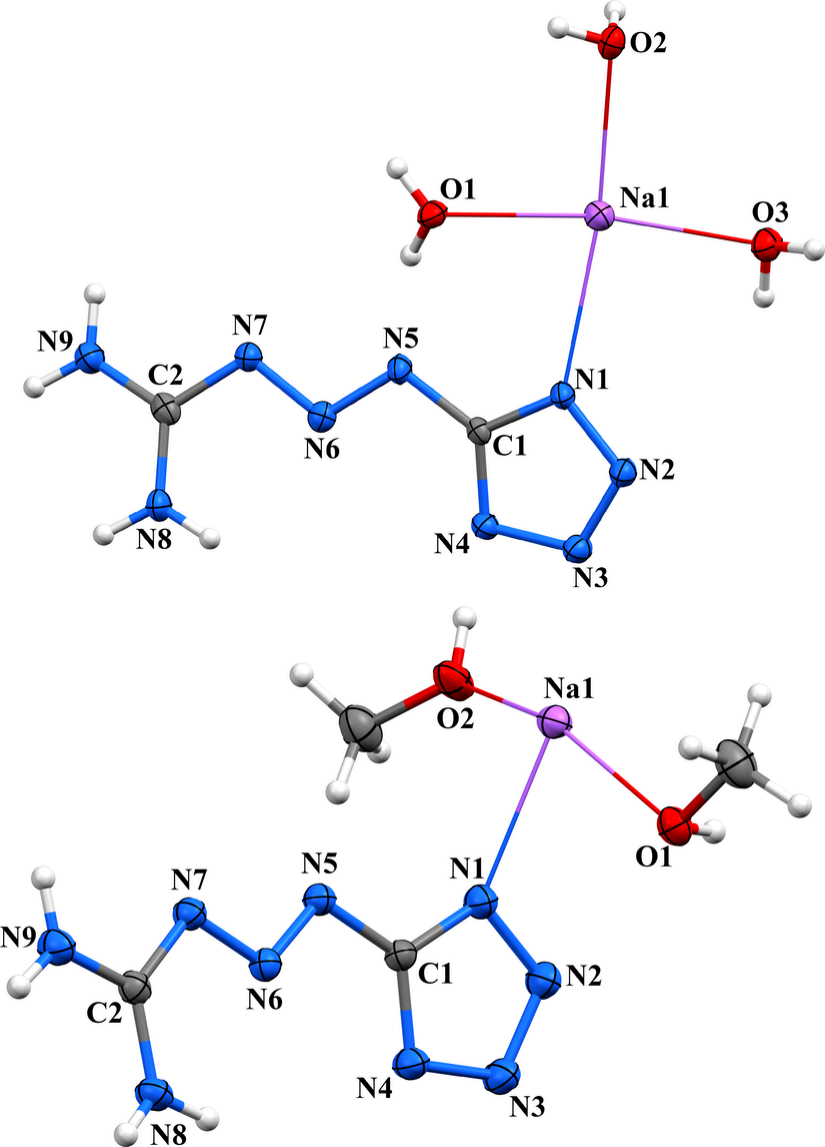
Monomeric structures of [MTX-1*·Na­(H_2_O)_3_]_*n*_ and [MTX-1*·Na­(MeOH)_2_]_*n*_. An ORTEP diagram, with a 50% probability level.

Crystal structure of **[MTX-1** * **·Na­(MeOH)**_**2**_ **]**_***n***_ exhibits structural changes in the coordination polymer upon replacing water with methanol as the solvent (Figure 8, Figure S18, and Scheme 1). The sodium cation retains a coordination number of 6 and adopts a distorted tetragonal bipyramidal geometry. However, in this case, the sodium cation is coordinated by **MTX-1** * molecules from the parallel layers. Unlike the linear chain observed in the structure with water, this crystal motif resembles a zigzag chain along the *b*-axis. (1D). This configuration promotes the formation of π–π-stacking interactions between parallel-oriented **MTX-1** * ligands from adjacent chains.

Crystal packages of **(MTX-1** * **)·[MTX-1** * **·Sr­(H**_**2**_ **O)**_**6**_ **]**_***n***_ and **(MTX-1** * **)·[MTX-1** * **·Ba­(H**_**2**_ **O)**_**6**_ **]**_***n***_ complexes are isostructural and have similar lattice parameters (Table S4). Superimposing these structures reveals only minor differences (Figure S13). Both complexes form 1D coordination polymers constructed through the linkage of monomeric fragments by two bridging water molecules (Figure 9, Figure S19, and Scheme 1). The strontium and barium cations exhibit a coordination number of 7, corresponding to a monocapped trigonal prism geometry. It should be noted, that each cation is coordinated by six water molecules and one **MTX-1** * anion, while the second **MTX-1** * anion remains solvent separated.

**Figure 9:**
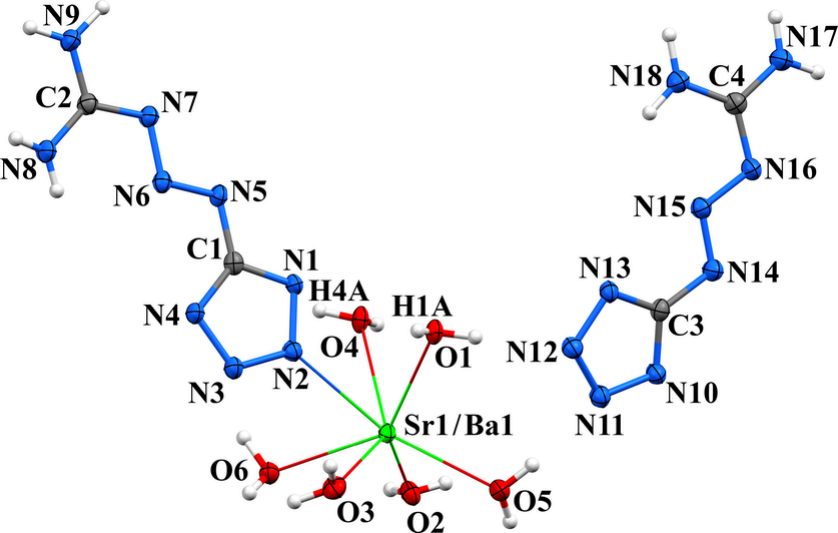
Monomeric structures of (MTX-1*)·[MTX-1*·Sr­(H_2_O)_6_]_*n*_ and (MTX-1*)·[MTX-1*·Ba­(H_2_O)_6_]_*n*_. Solvent molecules (H_2_O) have been omitted for the sake of clarity. An ORTEP diagram, with a 50% probability level. Symmetry codes: (A) 1–*x*, *y*, 1/2–*z*.

The π–π stacking interactions in these structures differ significantly from those in previous complexes. The coordinated **MTX-1** * anion participates in π–π stacking with the heads and tails of four adjacent uncoordinated molecules, following a “head-to-head” and “tail-to-tail” pattern. Uncoordinated water molecules are also present in the crystal lattice.

### 2D Coordination Polymers: [MTX-1*·K­(H_2_O)]_*n*_ and [MTX-1*·Rb­(H_2_O)]_*n*_

Crystal packing motif of **[MTX-1** * **·K­(H**_**2**_ **O)]**_***n***_ is, in contrast to the previous complexes, a 2D coordination polymer, where the layers are constructed from interconnected linear chains (Figure 10, Figure S20, and Scheme 1). The potassium cation exhibits a coordination number of 9, corresponding to a distorted monocapped square antiprism geometry. In this packing arrangement, the **MTX-1** * ligands are aligned parallel to each other. The unit cell contained voids that are occupied by free water molecules.

**Figure 10:**
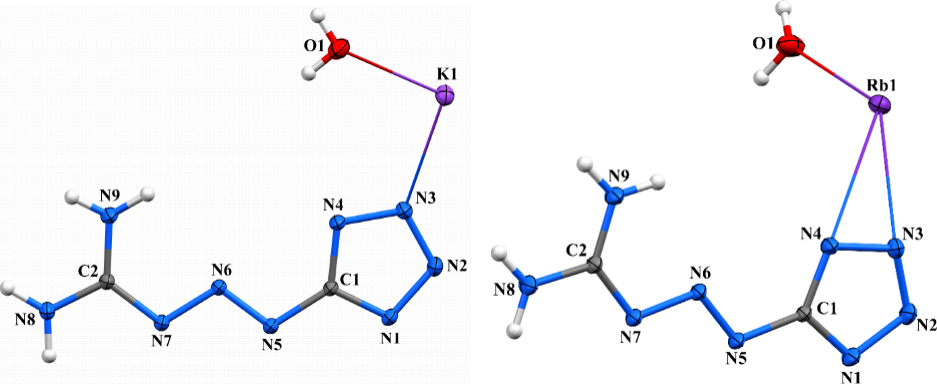
Monomeric structures of [MTX-1*·K­(H_2_O)]_*n*_ and [MTX-1*·Rb­(H_2_O)]_*n*_. Solvent molecules (H_2_O) are omitted for clarity. An ORTEP diagram, with a 50% probability level.

Rb complex forms a 2D coordination polymer (Figure 10, Figure S21, and Scheme 1), with the zigzag chains being interconnected via bridging **MTX-1** * anions. The packing contains voids filled by water molecules. The rubidium cation exhibits a coordination number of 10, corresponding to a distorted bicapped square antiprism geometry. In this packing arrangement, the opportunities for π–π stacking are reduced due to the specific mutual orientation of the deprotonated **MTX-1** * ligands: the heads are parallel to each other with a slight relative shift, while the tails are oriented in opposite directions.

### 3D Coordination Polymer

Crystal structure of **[MTX-1** * **·Cs­(H**_**2**_ **O)]**_***n***_ forms a 3D coordination polymer. The three-dimensional structure is constructed through the connection of chains and layers by bridging **MTX-1** * anions (Figure 11, Figure S22, and Scheme 1). The cesium cation exhibits a coordination number of 10, with geometry of a distorted bicapped square antiprism.

**Figure 11:**
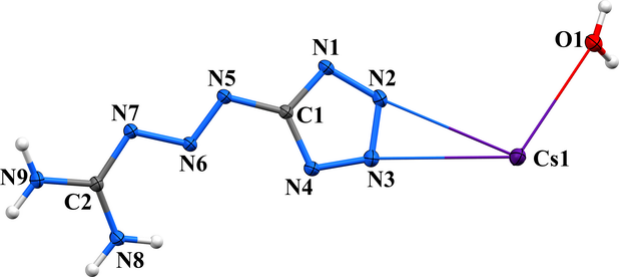
Monomeric structure of [MTX-1*·Cs­(H_2_O)]_*n*_. On the left picture, a solvent molecule (H_2_O) is omitted for clarity. An ORTEP diagram, with a 50% probability level.

Unlike the 2D structures (K and Rb), this complex lacks non-coordinated solvent molecules. Notably, this crystal has the highest density in the series (2.403 g/cm^3^) (Table S5).

A trend emerges when analyzing the ratio of the effective ionic radius to the charge of the ion (Table 1), providing insight into the relative preference for coordination with either water molecules or **MTX-1** * anions. As shown, Mg^2+^ exhibits the lowest *r*_eff_/charge value and is fully coordinated by water molecules. In contrast, Ca^2^^+^, with a slightly higher ratio, is already capable to coordinate **MTX-1** * ligand. As the *r*_eff_ increases further, K^+^ becomes able to participate in the coordination of multiple chains, forming layered structures, whereas Ba^2^^+^, despite having a similar *r*_eff_, prefers coordination with water molecules.

### Investigation of π–π Stacking Interactions

The study of intermolecular interactions in crystals enables the investigation of weak chemical forces, which, in turn, leads to a better understanding of the physicochemical properties of the substance. Among these, π–π stacking—a specific type of noncovalent interaction involving π-conjugated systems—plays a crucial role in molecular packing and stability. We aim to quantitatively evaluate the energy of such interactions based on the topological analysis of the electron density (ED). According to the geometric analysis of interlayer distances between deprotonated **MTX-1** * ligands in Li–Ba crystals, π–π stacking interactions are expected to be present in all of them. Figure 12 illustrates the relative orientations of **MTX-1** * ligands involved in it.

**Figure 12:**
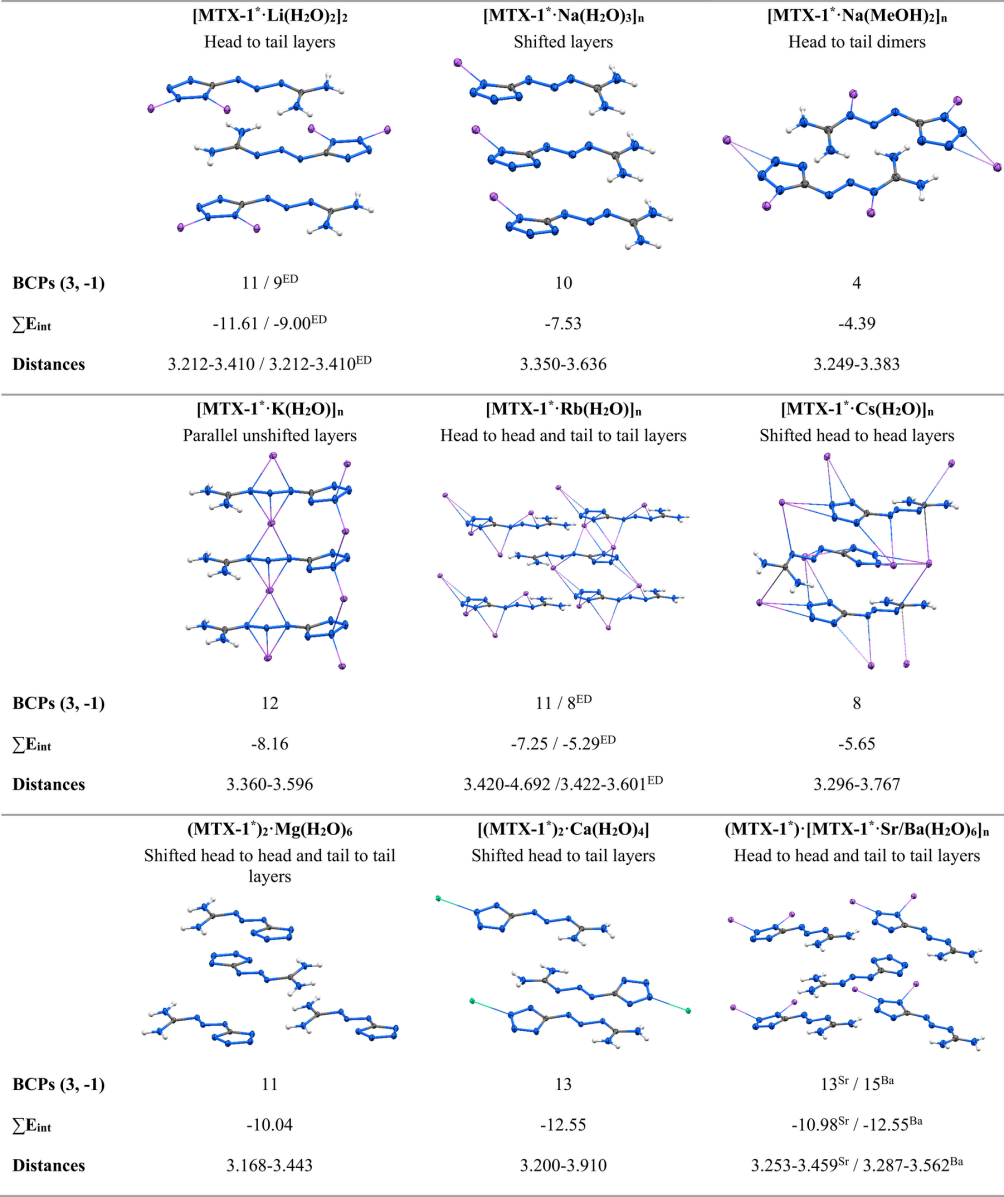
Different relative orientations of MTX-1* ligands in crystal Li–Ba during π–π stacking interactions. Solvent molecules and other atoms, except that coordinated ions are omitted for clarity. Amount of BCPs (3, −1), total interaction energies *E*_int_ (kcal/mol) of all of π–π stacking interactions per one MTX-1* ligand, and corresponding distances (Å).

Crystals **[MTX-1** * **·Li­(H**_**2**_ **O)**_**2**_ **]**_**2**_ and **[MTX-1** * **·Rb­(H**_**2**_ **O)]**_***n***_ were of sufficiently high quality to enable high-resolution X-ray diffraction experiments followed by multipole refinement (marked below as ED). The static deformation electron density distribution map for the **MTX-1** * anion in ^**ED**^ **[MTX-1** * **·Li­(H**_**2**_ **O)**_**2**_ **]**_**2**_ is shown on Figure S23.

The electron density distribution closely resembles that previously described for the tetrazene molecule.^41^ The accumulation of the electron density along the covalent bonds between atoms indicates strong π-conjugation within both the tetrazole and the guanidine fragments.

Topological analysis of the electron density distribution was performed within the Bader’s “Atoms in Molecules” theory^42^ using both experimental data **(** ^**ED**^ **[MTX-1** * **·Li­(H**_**2**_ **O)**_**2**_ **]**_**2**_, ^**ED**^ **[MTX-1** * **·Rb­(H**_**2**_ **O)]**_***n***_ **)** and theoretical calculations (Li–Ba complexes) at the M062X-D3­(BJ)/def2-TZVPD level of theory. For each crystal, a cluster was selected in which the **MTX-1** * ligand exhibited the maximum number of neighboring molecules. The geometry was then fixed, and a single-point calculation was carried out. The intermolecular interaction energies *E*_int_ were estimated using Espinosa’s correlation equation^43^ at the bond critical points (3, −1) (BCP) corresponding to π–π stacking interactions. The individual energies were summed, and the corresponding interaction distances were recorded (Figure 12 and Table S5). Molecular graphs of the selected clusters are shown in Figure 13 and Figures S24–S33.

**Figure 13:**
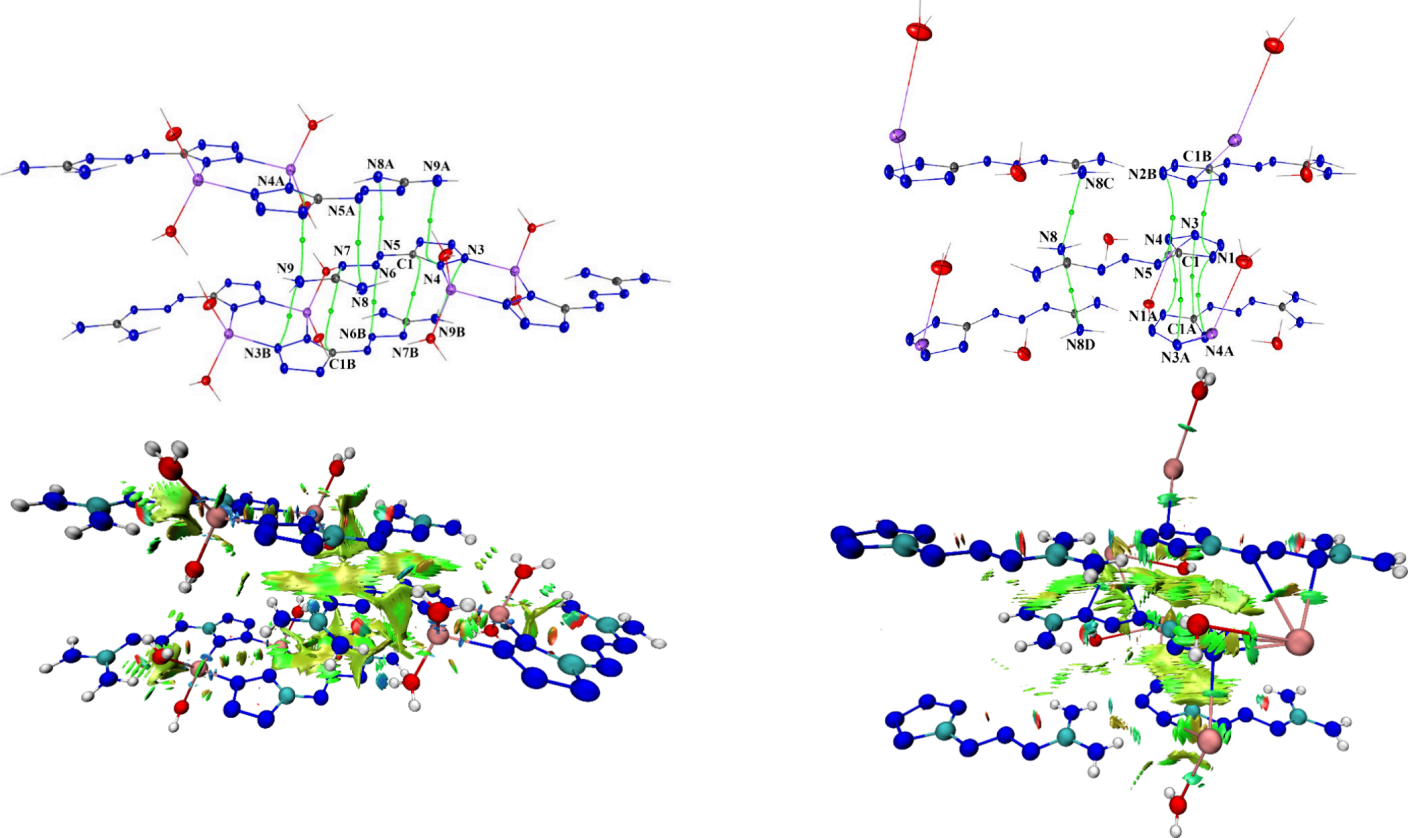
Molecular graphs derived from experimental data and relative theoretical NCI plots of gradient isosurfaces (*s* = 0.5 au) for a key fragment of the unit cell of ^ED^[MTX-1*·Li­(H_2_O)_2_]_2_ (left) and ^ED^[MTX-1*·Rb­(H_2_O)]_*n*_ (right). Thermal ellipsoids are drawn at the 50% probability level, and only BCPs (3, −1) and bond paths involved in π–π stacking interactions are shown for clarity. Symmetry codes: ^ED^[MTX-1*·Li­(H_2_O)_2_]_2_: (A) −*x*, 1–*y*, 1–*z*; (B) 1–*x*, 1–*y*, 1–*z*. ^ED^[MTX-1*·Rb­(H_2_O)]_*n*_: (A) 1–*x*, 1–*y*, 1–*z*; (B) 1–*x*, 2–*y*, 1–*z*; (C) 2–*x*, 2–*y*, 1–*z*; (D) 2–*x*, 1–*y*, 1–*z*. The surfaces are colored on a blue-green-red (BGR) scale, ranging from −0.04 to 0.02 au.

As shown in Figure 12, the total energies of π–π stacking interactions per **MTX-1** * ligand differ by 2.61 and 1.96 kcal/mol for ^**ED**^ **[MTX-1** * **·Li­(H**_**2**_ **O)**_**2**_ **]**_**2**_ and ^**ED**^ **[MTX-1** * **·Rb­(H**_**2**_ **O)]**_***n***_, respectively, in comparison with theoretical calculations. This difference arises from the localization of two and three BCPs (3, −1) fewer in ^**ED**^ **[MTX-1** * **·Li­(H**_**2**_ **O)**_**2**_ **]**_**2**_ and ^**ED**^ **[MTX-1** * **·Rb­(H**_**2**_ **O)]**_***n***_, respectively, compared to the theoretical data. It is worth noting that, in the molecular graph of Rb complex, a BCP (3, −1) corresponding to the N48···N84 contact (Figure S28) was localized at the distance of 4.692 Å, which significantly exceeds the sum of the van der Waals radii of nitrogen atoms (3.2 Å).^44^ Consequently, the energy of this weak interaction is only −0.03 kcal/mol, and it is not observed in the experimental data. Therefore, the range of distances at which bond critical points could be localized is 3.168–4.692 Å.

The electron density involved in the formation of π–π stacking is highly delocalized, as clearly demonstrated by the noncovalent interaction (NCI) plots (Figure 13). NCI plots for the remaining compounds are provided in the Supporting Information (Figures S25–S2 and S29–S33). It is worth noting that the smallest absolute value of the π–π stacking interaction.

Energy (−4.39 kcal/mol) is observed for **[MTX-1** * **·Na­(MeOH)**_**2**_ **]**_***n***_, where the **MTX-1** * anion forms dimeric stacks (Figure 12). The strongest interaction, with the largest absolute energy value (−12.55 kcal/mol), corresponds to **[(MTX-1** * **)**_**2**_ **·Ca­(H**_**2**_ **O)**_**4**_ **]** and **(MTX-1** * **)·[MTX-1** * **·Ba­(H**_**2**_ **O)**_**6**_ **]**_***n***_. Although complex **[MTX-1** * **·Rb­(H**_**2**_ **O)]**_***n***_ exhibits a structural motif similar to that of **(MTX-1** * **)·[MTX-1** * **·Sr­(H**_**2**_ **O)**_**6**_ **]**_***n***_ and **(MTX-1** * **)·[MTX-1** * **·Ba­(H**_**2**_ **O)**_**6**_ **]**_***n***_, the increased interplanar distances in **[MTX-1** * **·Rb­(H**_**2**_ **O)]**_***n***_ result in a lower π–π interaction energy. Notably, no clear correlation is observed between the dimensionality of the coordination polymer and the magnitude of the π–π stacking energy.

## Experimental Section

### Materials and Methods

**Caution!** Due to the fact that energetic tetrazole compounds could be to some extent unstable and sensitive against outer stimuli, proper safety precautions should be taken when handling the materials. Especially dry samples of **MTX-1** are able to explode under the influence of impact or friction. Lab personnel and the equipment should be properly grounded, and protective equipment like grounded shoes, leather coat, Kevlar gloves, ear protection, and face shield is recommended for the handling of any energetic material. Friction, impact sensitivity, Koenen, and detonation tests must be performed by certified personnel only.

The setups and descriptions of differential thermal analysis, friction and impact sensitivity tests, Koenen test, detonation test, elemental analysis, color purity, Fourier-transform infrared spectroscopy, X-ray crystallography, DFT calculations, and QTAIM analysis, as well as safety precautions and synthetic details, are provided in the Supporting Information.

## Conclusions

Reactions of **MTX-1** with the hydroxides of alkali or alkaline earth metals produce coordination compounds with various compositions. These hydrated complexes are insensitive to mechanical stimuli, such as impact and friction, and are insensitive to intense heat under high confinement. These stable, hydrated complexes could be used for intense flame coloring in pyrotechnics. High-yield recovery of insoluble **MTX-1** is possible when the complexes are treated with acetic acid, which can be used for the safe transportation, stockpiling, and on-site generation of **MTX-1**. The experimentally observed high stability and structural diversity were compared and explained by theoretical calculations. Most intact complexes bear ions with the smallest effective ion radii (Mg^2^^+^ and Ca^2^^+^), followed by Li^+^, forming mononuclear or dimeric structures in the solid state. Other types of 1D (Na^+^, Sr^2^^+^, and Ba^2^^+^), 2D (K^+^ and Rb^+^), and 3D (Cs^+^) coordination polymers were identified for the remaining metals. A detailed topological analysis of electron density, as well as a quantitative estimation of π–π stacking interactions reflected in total interaction energies, correlates with the stability of the complexes. Solvent exchange combined with the use of higher-valent metals opens broad opportunities for future research in this field.

## Supplementary Material





## Notes

(XYZ)
Preparation details, spectra and spectral parameters, crystallography, and calculations (PDF)
