# Supplementary material for: Safe Stockpiling of the MTX‑1 Primary Explosive in Alkali or Alkaline Earth Metal Complexes and Coordination Polymers
Source: Inorg Chem. 2026 Feb 18;65(8):4420–9. doi: 10.1021/acs.inorgchem.5c04892 (PMC12958285; doi:10.1021/acs.inorgchem.5c04892)
Supplement: Supplementary file 2 [file ic5c04892_si_002.pdf]

Supporting Information

The Safe Stockpiling of MTX-1 Primary Explosive in Alkali or Alkaline Earth Metal Complexes and Coordination Polymers

Maksim A. Samsonov,[a] Jakub Mikuláštík,[b] Robert Matyáš,*[b] Aleš Růžička,*[a]

[a]Department of General and Inorganic Chemistry, Faculty of Chemical Technology, University of Pardubice, Studentská 573, CZ-532 10, Pardubice, Czech Republic

[b]Institute of Energetic Materials, Faculty of Chemical Technology, University of Pardubice, Studentská 573, CZ-532 10, Pardubice, Czech Republic

*Corresponding authors e-mail: robert.matyas@upce.cz, ales.ruzicka@upce.cz

Table of Contents

Materials and Methods

Differential Thermal Analysis……S3

Friction and impact sensitivity……S3

Koenen test……S3

Detonation test……S4

Elemental analysis……S4

Color purity……S4

Fourier Transform Infrared Spectroscopy……S4

X-ray Crystallography……S4

DFT calculations and QTAIM analysis……S5

Safety Precautions……S6

Synthesis

MTX-1……S6

Lithium salt of MTX-1 - [MTX-1*·Li$(H_2O)_2]_2$……S6

Sodium salt of MTX-1 - [MTX-1*·Na$(H_2O)_3]_n$……S6

Sodium salt of MTX-1 (methanol solvate) - [MTX-1*·Na$(MeOH)_2]_n$……S7

Potassium salt of MTX-1 - [MTX-1*·K$(H_2O)]_n$……S7

Rubidium salt of MTX-1 - [MTX-1*·Rb$(H_2O)]_n$……S7

Cesium salt of MTX-1 - $[MTX\text{-}1^{*}\cdot Cs(H_2O)]_n$....S7
Magnesium salt of MTX-1 - $(MTX\text{-}1^{*})_2\cdot Mg(H_2O)_6$....S8
Calcium salt of MTX-1 - $[(MTX\text{-}1^{*})_2\cdot Ca(H_2O)_4]$....S8
Strontium salt of MTX-1 - $(MTX\text{-}1^{*})\cdot[MTX\text{-}1^{*}\cdot Sr(H_2O)_6]_n$....S8
Barium salt of MTX-1 - $(MTX\text{-}1^{*})\cdot[MTX\text{-}1^{*}\cdot Ba(H_2O)_6]_n$....S8
Preparation of single crystalline material....S9
Dehydration of MTX-1* salts....S9
Recovery of MTX-1 from $[MTX\text{-}1^{*}\cdot Na(H_2O)_3]_n$....S9
Thermal stability screening (Modified UN Test 3(c)....S9
Long-term stability assessment....S9
Experimental data
FTIR and Raman spectrum....S10
Sensitivity data....S20
Detonation test....S21
X-ray data....S23
Theoretical data....S29
References....S44

EXPERIMENTAL SECTION

Materials and Methods

Caution!

Due to the fact that energetic tetrazole compounds could be to some extend unstable against outer stimuli, proper safety precautions should be taken when handling the materials. Especially dry samples are able to explode under the influence of impact or friction. Lab personnel and the equipment should be properly grounded and protective equipment like grounded shoes, leather coat, Kevlar gloves, ear protection and face shield is recommended for the handling of any energetic material.

Differential Thermal Analysis

Thermal analysis was performed using a DTA 550Ex differential thermal analyser (OZM Research, Czech Republic). The samples were tested in open glass microtest tubes in contact with air at a heating rate of 10 °C min^{-1}. The sample weights were 50 mg.

Friction and impact sensitivity

The sensitivity to friction was determined using a BAM FSA-12 type FSA-12. The test sets consisted of porcelain plates BFST Pt 100 25 × 25 mm and porcelain BFST Pn 200 pegs. The measurement apparatus and related supplies were manufactured by OZM Research (Hrochův Týnec, Czech Republic). Probit analysis was used for measuring and evaluating.[1] Five energy levels were measured for 15 trials for each friction force level.

The sensitivity to impact was measured using a Kast fall hammer. The measurement apparatus and related supplies were manufactured by OZM Research (Hrochův Týnec, Czech Republic). We used a 0.5 and 1 kg hammer for the measurement. The testing sets comprised steel guide rings BFH-SR and steel cylinders BFH-SC. A probit analysis was used to measure and evaluate the data.[1] Five energy levels were measured for 15 trials for each energy level. Friction and impact sensitivity curves for reference explosives mercury fulminate (MF), pentaerythritol tetranitrate (PETN), and 1,3,5-trinitro-1,3,5-triazinane (RDX) were taken from our previous work.[2]

Koenen test

The Koenen test apparatus was produced by Explosia, a.s. (Pardubice, Czech Republic), and the steel test tube assembly was produced by OZM Research (Hrochův Týnec, Czech Republic). The tube was filled with 27 cm^3 of the sample by pressing, and then closed with a closing device with an orifice 2 mm in diameter. The test tube assembly was then placed in the heating device, and the burners were ignited. The measuring apparatus, related supplies, and measurement methodology were in compliance with the regulations.[3]

Detonation test

The detonation behavior of the anhydrous sodium salt of MTX-1 was investigated. The powdered sample (87.4 g) was lightly pressed into a paper tube (inner diameter 32 mm) to a loading density of 0.475 $g{\cdot}cm^{-3}$. The charge was initiated using a 21.3 g plastic explosive, Semtex 1A as a booster, and a standard electric detonator. The reaction front velocity was monitored simultaneously by three independent methods: (i) a series of ionization probes connected to a digital oscilloscope, (ii) discrete glass optical fibers affixed to the charge exterior, and (iii) a continuous plastic fiber optic probe (FOP) inserted along the central axis of the charge. The signals from both optical methods were recorded by an OZM Optimex 64 system. Photo of the setup before and after the test is included in the Supplementary Information section (Fig. S13-S14).

Elemental analysis

Elemental analysis was carried out using a UNICUBE automatic elemental analyzer (Elementar, Germany) on 1−2 mg samples.

Color purity

The color purity of samples in flame was measured using a fiber spectrometer Ocean FX (Ocean Optics), and the spectra were evaluated using OceanView 2.0.15. For the measurement, a small amount of the sample was placed on a spatula and introduced into the flame of a gas burner.

Fourier Transform Infrared Spectroscopy

Infrared spectra were collected using a Nicolet iS50 FT-IR spectrometer (Thermo Fisher, USA) with an iS50ATR diamond. Measurement parameters were the spectral regions of 4000−400 cm^{-1}, the resolution of 4 cm^{-1}, and 64 scans. The Raman spectra were measured using a Nicolet

iS50 Raman module with the following settings: 1064 nm excitation laser at 100–500 mW power range, spectral region 4000–100 cm−1, resolution 4 cm−1 and 128 scans. Measured FTIR and Raman spectra are included in the Supplementary Information section (Fig. S1-S10).

X-ray Crystallography

Full-sets of diffraction data for all of compounds were collected at 150(2)K with a Bruker D8-Venture diffractometer equipped with Mo (Mo/Kα radiation; λ = 0.71073 Å) microfocus X-ray (IμS) source, Photon CMOS detector and Oxford Cryosystems cooling device was used for data collection. The frames were integrated with the Bruker SAINT software package using a narrowframe algorithm. Data were corrected for absorption effects using the Multi-Scan method (SADABS). Obtained data were treated by XT-version 2018/2 and SHELXL-2018/3 software implemented in APEX4 v2022.1-1 (Bruker AXS) system.[4] Hydrogen atoms were localized on a difference Fourier map.

The multipole refinement was carried out within the Hansen–Coppens formalism[5] using the MoPro v18 program package.[6] Before the refinement, C–H bond distances were normalized to the values obtained in neutron diffraction analyses.[7] The level of the multipole expansion was hexadecapole for the Rb1, octupole for all other non-hydrogen atoms, and one dipole for hydrogen atoms. The refinement of compounds **^{ED}[MTX-1*·Li(H_2O)$_2$]$_2$**, **^{ED}[MTX-1*·Rb(H_2O)]$_n$** was carried out against F and converged to R = 0.0172, wR = 0.0537, GOF = 2.268 for 9236 merged reflections with *I>2σ(I) and* R = 0.0391, wR = 0. 0714, GOF = 1.026 for 10616 merged reflections with *I>2σ(I)* respectively. All bonded pairs of atoms satisfy the Hirshfeld rigid-bond criteria.[8] Analysis of topology of the experimental *ρ*(***r***) function was carried out using the MoProViewer program.[9] The residual electron densities around Rb is 1.86e, however, attempts to reduce this value by varying the parameters when taking absorption into account have not been successful.

Crystallographic data for structural analysis has been deposited with the Cambridge Crystallographic Data Centre, CCDC nos. 2482905-2482914. Copies of this information may be obtained free of charge from The Director, CCDC, 12 Union Road, Cambridge CB2 1EY, UK (fax: +44-1223-336033; e-mail: deposit@ccdc.cam.ac.uk or www: http://www.ccdc.cam.ac.uk).

DFT calculations and QTAIM analysis

All the calculations were performed with the Gaussian 16 program[10] at the M062X-D3(BJ)/def2-TZVPD level of theory.[11-13] To estimate of intermolecular interactions around **MTX-**

1* anion a cluster was selected in which the **MTX-1*** exhibited the maximum number of neighboring molecules. The geometry was then fixed, and a single-point calculation was carried out. The NCI analysis[14] was done in Multiwfn v.3.8 [15] and plots visualized in VMD 1.9.4a53.[16] The topological analysis of the theoretical function $\rho(\boldsymbol{r})$ was performed using the AIMALL 19.10.12 program package.[17]

Safety Precautions

MTX-1 is a sensitive primary explosive. The synthesis and handling of MTX-1 is hazardous, and it is necessary to strictly follow all the safety precautions for handling primary explosives even when working with small quantities!

Synthesis

MTX-1

MTX-1 was prepared from tetrazene[18] according to the procedure described by Fronabarger and Williams.[19, 20] DTA (10 °C min^{-1}, onset): 195 °C (exo). Elemental analysis: calcd. (%) for $C_2H_5N_9$: C 15.49, H 3.25, N 81.27. Found: C 15.01, H 3.43, N 78.26.

Lithium salt of MTX-1 - [MTX-1*·Li(H$_2$O)$_2$]$_2$

MTX-1 (250 mg, 1.61 mmol) was dissolved in a solution of lithium hydroxide monohydrate (68 mg, 1.62 mmol) in 5 mL of methanol and 1 mL of water. The solvent was evaporated in vacuo and the crude yellow-orange product was recrystallized from water/ethanol mixture (1:1). The resulting orange crystals were collected by filtration and dried (223 mg, 70.2 %). DTA (10 °C min^{-1}, onset): 198 °C (endo), 229 (exo). Elemental analysis: calcd. (%) for $C_2H_8N_9LiO_2$: C 12.19, H 4.09, N 63.97, Li 3.52, O 16.24. Found: C 12.33, H 3.97, N 63.77.

Sodium salt of MTX-1 - [MTX-1*·Na(H$_2$O)$_3$]$_n$

Method A (Evaporation): MTX-1 (2.0 g, 12.9 mmol) was stirred in a solution of sodium hydroxide (0.51 g, 12.8 mmol) in 25 mL of methanol and 10 mL of water. After 10 min, the mixture was filtered, the filtrate was evaporated in vacuo, and the solid was recrystallized from a water/ethanol mixture (5:1) to yield orange crystals after collection by filtration (2.57 g, 86.2 %).

Method B (Precipitation): MTX-1 (2.5 g, 16.1 mmol) was dissolved in a solution of sodium hydroxide (0.73 g, 18.3 mmol) in 10 mL of water. To this solution, 50 ml of acetone was added

dropwise over 10 minutes, resulting in the formation of a precipitate. After the addition of acetone, the suspension was stirred for 30 min, and the precipitated solid was collected by filtration to yield a yellow-orange product. (3.45 g, 92.6 %).

DTA (10 °C min^{-1}, onset): 87 °C (endo), 128 °C (endo), 157 °C (endo), 248 °C (exo). Elemental analysis: calcd. (%) for $C_2H_{10}N_9NaO_3$: C 10.39, H 4.36, N 54.54, Na 9.95, O 20.77. Found: C 10.42, H 4.27, N 55.33.

Sodium salt of MTX-1 (methanol solvate) - $[MTX\text{-}1^{*}\cdot Na(MeOH)_2]_n$

Crystals suitable for X-ray analysis were grown as yellow rods by dissolving dehydrated NaMTX-1* in a minimum amount of hot anhydrous methanol and allowing the solution to slowly cool to room temperature.

Potassium salt of MTX-1 - $[MTX\text{-}1^{*}\cdot K(H_2O)]_n$

MTX-1 (5.0 g, 32.2 mmol) was stirred in a solution of potassium hydroxide (2.18 g, 38.9 mmol) in 25 mL of methanol and 60 mL of water. After 10 min of stirring the solution was filtered, the solution was evaporated in vacuo and the yellow-orange product was recrystallized from the water/ethanol mixture (5:1) yielding orange crystals after collection by filtration (6.25 g, 84.6 %). DTA (10 °C min^{-1}, onset): 92 °C (endo), 170 °C (endo), 241 °C (exo). Elemental analysis: calcd. (%) for $C_2H_8N_9KO_2$: C 10.48, H 3.52, N 54.99, K 17.06, O 13.96. Found: C 10.74, H 3.46, N 57.83.

Rubidium salt of MTX-1 - $[MTX\text{-}1^{*}\cdot Rb(H_2O)]_n$

MTX-1 (1.60 g, 10.3 mmol) was dissolved in a mixture of 50% rubidium hydroxide solution (2 g, 9.76 mmol), 15 mL of methanol, and 5 mL of water. The mixture was stirred for 10 min, and after filtration, the solvent was evaporated in vacuo, and the yellow-orange product was recrystallized from the water/ethanol mixture (1:1) yielding orange crystals after collection by filtration (2.01 g, 74.8 %). DTA (10 °C min^{-1}, onset): 112 °C (endo), 235 °C (exo). Elemental analysis: calcd. (%) for $C_2H_8N_9RbO_2$: C 8.72, H 2.93, N 45.74, Rb 31.01, O 11.61. Found: C 8.77, H 2.78, N 46.39.

Cesium salt of MTX-1 - $[MTX\text{-}1^{*}\cdot Cs(H_2O)]_n$

MTX-1 (1.10 g, 7.09 mmol) was dissolved in a mixture of 50% cesium hydroxide solution (2g, 6.67 mmol), 15 mL of methanol, and 5 mL of water. The mixture was stirred for 10 min, and after filtration, the solvent was evaporated in vacuo, and the yellow-orange product was recrystallized from the water/ethanol mixture (1:1) yielding orange crystals after collection by filtration (1.94 g,

95.4 %). DTA (10 °C min^{-1}, onset): 134 °C, 224 °C (exo). Elemental analysis: calcd. (%) for $C_2H_6N_9CsO$: C 7.87, H 1.98, N 41.33, Cs 43.57, O 5.25. Found: C 7.70, H 1.91, N 41.0.

Magnesium salt of MTX-1 - $(MTX\text{-}1^*)_2{\cdot}Mg(H_2O)_6$

MTX-1 (1.93 g, 12.4 mmol) was suspended in 800 mL of water together with $MgCO_3.Mg(OH)_2$ (3.0 g, 35.6 mmol), and stirred for 24 h. The mixture was filtered, and the yellow filtrate was evaporated in vacuo. The product was recrystallized from water, yielding a yellow crystalline powder (2.27 g, 83.1 %). DTA (10 °C min^{-1}, onset): 163 °C (endo), 200 °C (exo). Elemental analysis: calcd. (%) for $C_4H_{20}N_{18}MgO_6$: C 10.90 H 4.58, N 57.22, Mg 5.52, O 21.79. Found: C 10.87 H 4.499, N 56.88.

Calcium salt of MTX-1 - $[(MTX\text{-}1^*)_2{\cdot}Ca(H_2O)_4]$

MTX-1 (1.93 g, 12.4 mmol) was suspended in 800 mL of water together with $Ca(OH)_2$ (3.1 g, 41.8 mmol), and stirred for 24 h. The mixture was filtered, and the yellow filtrate was evaporated in vacuo. The product was recrystallized from water to yield a yellow crystalline powder (2.02 g, 77.6 %). DTA (10 °C min^{-1}, onset): 206 °C (endo), 232 °C (exo). Elemental analysis: calcd. (%) for $C_4H_{16}N18CaO_4$: C 11.43, H 3.84, N 59.98, Ca 9.53, O 15.22. Found: C 11.21, H 3.616, N 58.80.

Strontium salt of MTX-1 - $(MTX\text{-}1^*){\cdot}[MTX\text{-}1^*{\cdot}Sr(H_2O)_6]_n$

MTX-1 (2.0 g, 12.9 mmol) was dissolved in a solution of $Sr(OH)_2.8H_2O$ (1.71 g, 6.43 mmol) in 300 mL of water. The resulting yellow solution was filtered, and water was evaporated in vacuo, and the yellow-orange powder was recrystallized from water, yielding beige crystalline powder (2.47 g, 70.8 %). DTA (10 °C min^{-1}, onset): 105 °C (endo), 209 °C (endo), 230 °C (exo). Elemental analysis: calcd. (%) for $C_4H_{24}N_{18}SrO_8$: C 8.90, H 4.48, N 46.69, Sr 16.23 O 23.70. Found: C 8.93, H 4.374, N 47.56.

Barium salt of MTX-1 - $(MTX\text{-}1^*){\cdot}[MTX\text{-}1^*{\cdot}Ba(H_2O)_6]_n$

MTX-1 (2.0 g, 12.9 mmol) was dissolved in a solution of $Ba(OH)_2.8H_2O$ (2.0 g, 6.34 mmol) and 300 mL of water. The resulting yellow solution was filtered, and water was evaporated in vacuo, and the yellow-orange powder was recrystallized from water, yielding yellow-orange crystalline powder (2.39 g, 62.9 %). DTA (10 °C min^{-1}, onset): 82 °C (endo), 155 °C (endo), 234 °C (exo). Elemental analysis: calcd. (%) for $C_4H_{24}N_{18}BaO_8$: C 8.15, H 4.10, N 42.76, Ba 23.29, O 21.17. Found: C 8.08, H 3.914, N 43.28.

Preparation of single crystalline material

Single crystals suitable for X-ray analysis were obtained for each MTX-1 salt by dissolving the compound in a minimum amount of water (methanol) to form a concentrated solution. The solutions were allowed to evaporate slowly under ambient conditions from plastic vials that were covered with perforated lids.

Dehydration of MTX-1* salts

The hydrates of Na, K, Rb, Cs, Sr, and Ba salts (approx. 2 g each) were heated in a drying oven at 140 °C for 30 minutes to achieve a constant weight. The complete removal of crystal water was confirmed by Karl Fischer titration. The corresponding hydrates of Li, Mg, and Ca could not be dehydrated without decomposition.

Recovery of MTX-1 from $[MTX\text{-}1^{*}\cdot Na(H_2O)_3]_n$

A solution of sodium salt MTX-1* trihydrate (5.0 g, 21.6 mmol) in 75 mL of water was acidified by the dropwise addition of 10 mL aqueous solution of acetic acid (1.44 mL, 24.0 mmol), which caused MTX-1 to precipitate. The resulting suspension was stirred for 30 minutes, after which the solid was collected by vacuum filtration (3.11 g, 92.7 %). DTA (10 °C min^{-1}, onset): 194 °C (exo). The identity of the recovered product was confirmed by FTIR, Raman spectroscopy, and DTA, yielding data consistent with the original MTX-1 starting material (see Figure S1).

Thermal stability screening (Modified UN Test 3(c)[3])

The thermal stability was screened based on the UN Test 3(c). The test was conducted using DTA 550Ex differential thermal analyzer (OZM Research, Czech Republic). A 100 mg of $[MTX\text{-}1^{*}\cdot Na(H_2O)_3]_n$ sample was heated at 75°C for 48 hours with monitoring the temperature difference between the sample and reference. No exothermic reaction observed; the temperature difference remained constant with a negligible maximum deviation of 0.05°C.

Long-term stability assessment

The long-term chemical stability of $\mathbf{[MTX\text{-}1^{*}\cdot Na(H_2O)_3]_n}$ was evaluated by comparing a freshly prepared sample with samples stored in closed plastic containers under ambient laboratory conditions for 6 months and 2 years, respectively. The physicochemical integrity of the aged samples was verified using elemental analysis, differential thermal analysis (DTA), and vibrational spectroscopy (FTIR, Raman). Comparison of elemental analysis and DTA data (Table S1), along with vibrational spectra (Figure S12) confirmed no physicochemical changes in the aged samples.

FTIR and Raman spectrum

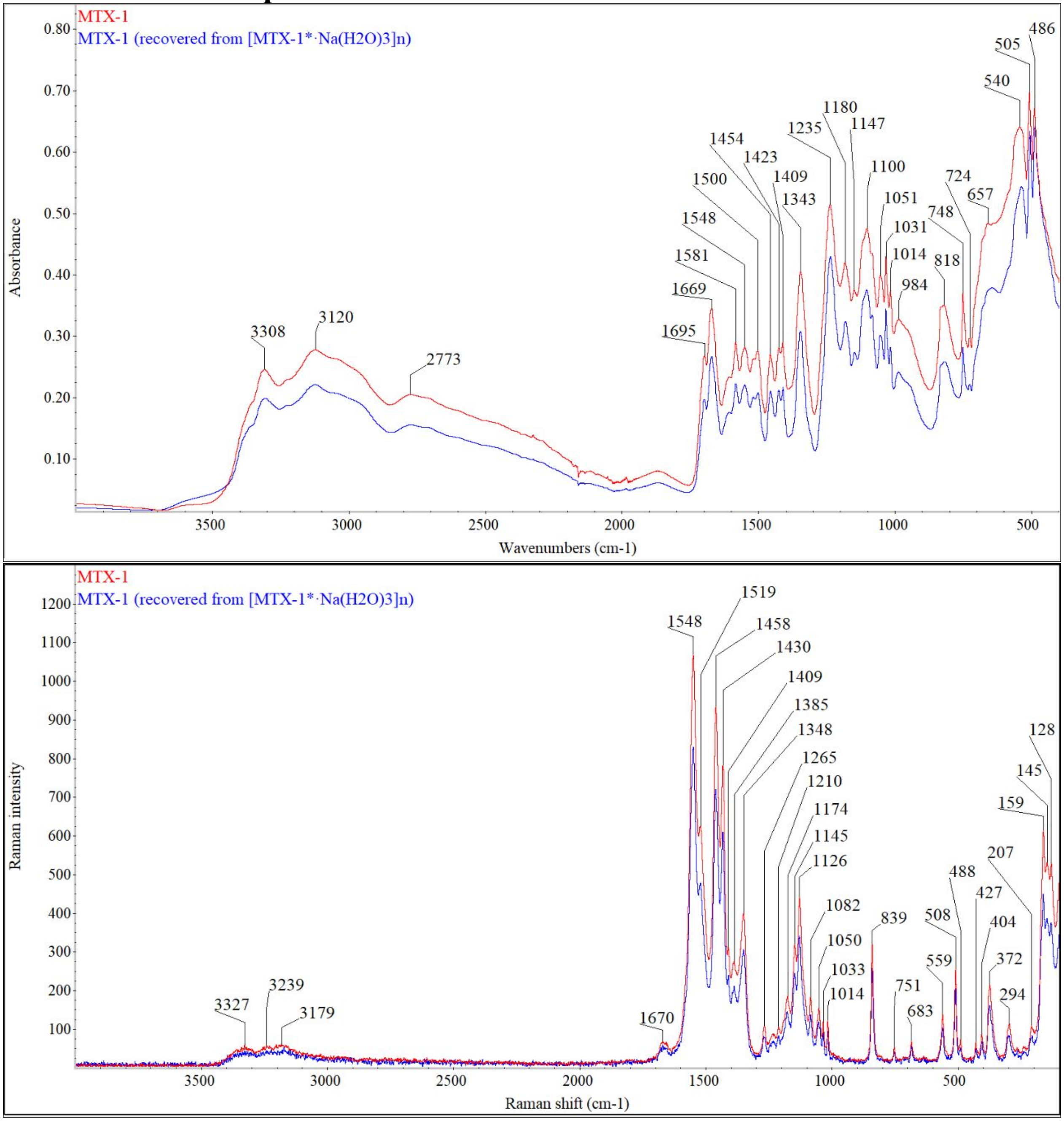

Figure S1. FTIR (top) and Raman (bottom) spectra of **MTX-1** as prepared and **MTX-1** recovered from **[MTX-1*·Na(H_2O)$_3$]$_n$**.

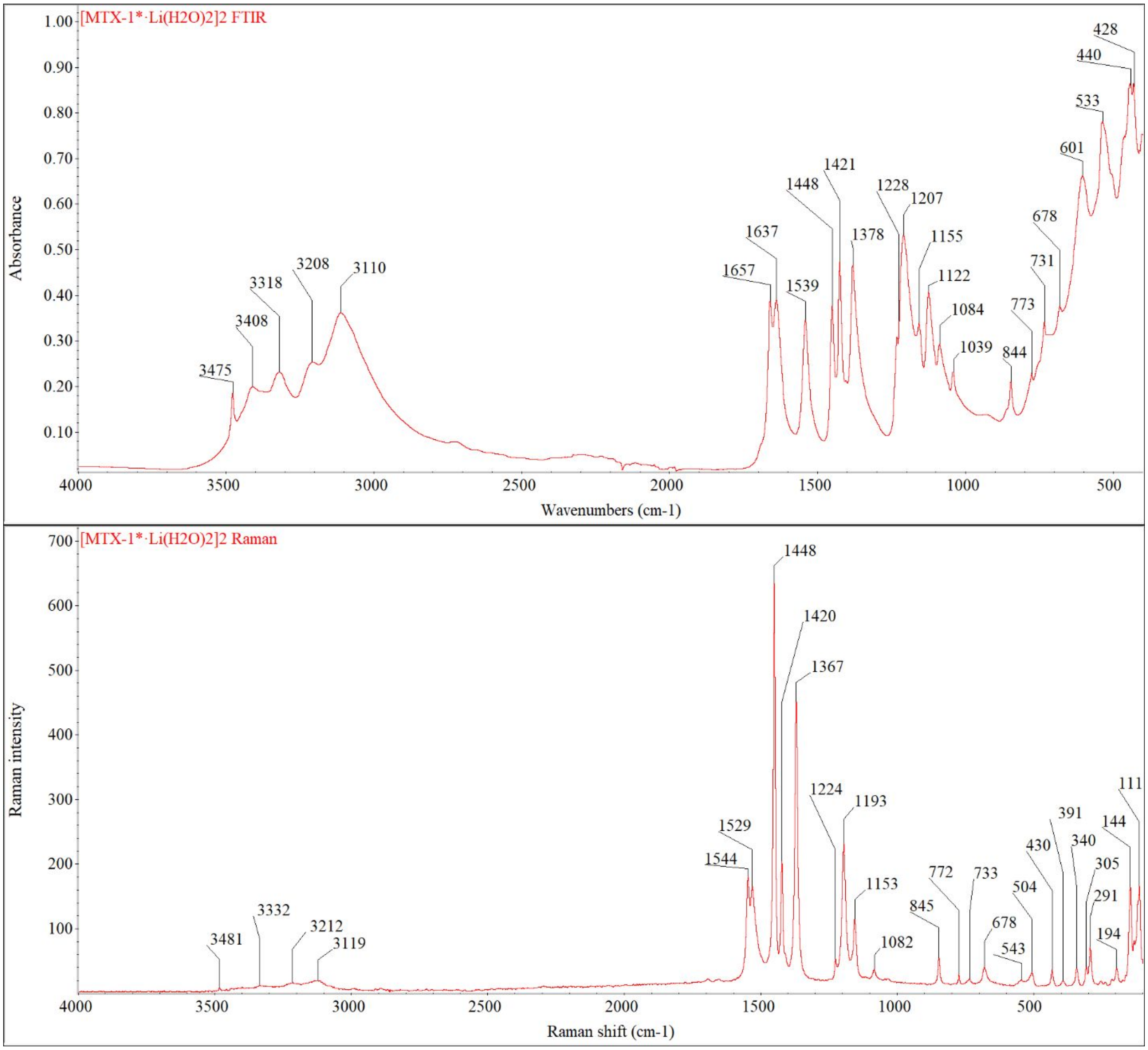

Figure S2. FTIR (top) and Raman (bottom) spectra of **[MTX-1*·Li(H_2O)$_2$]$_2$**.

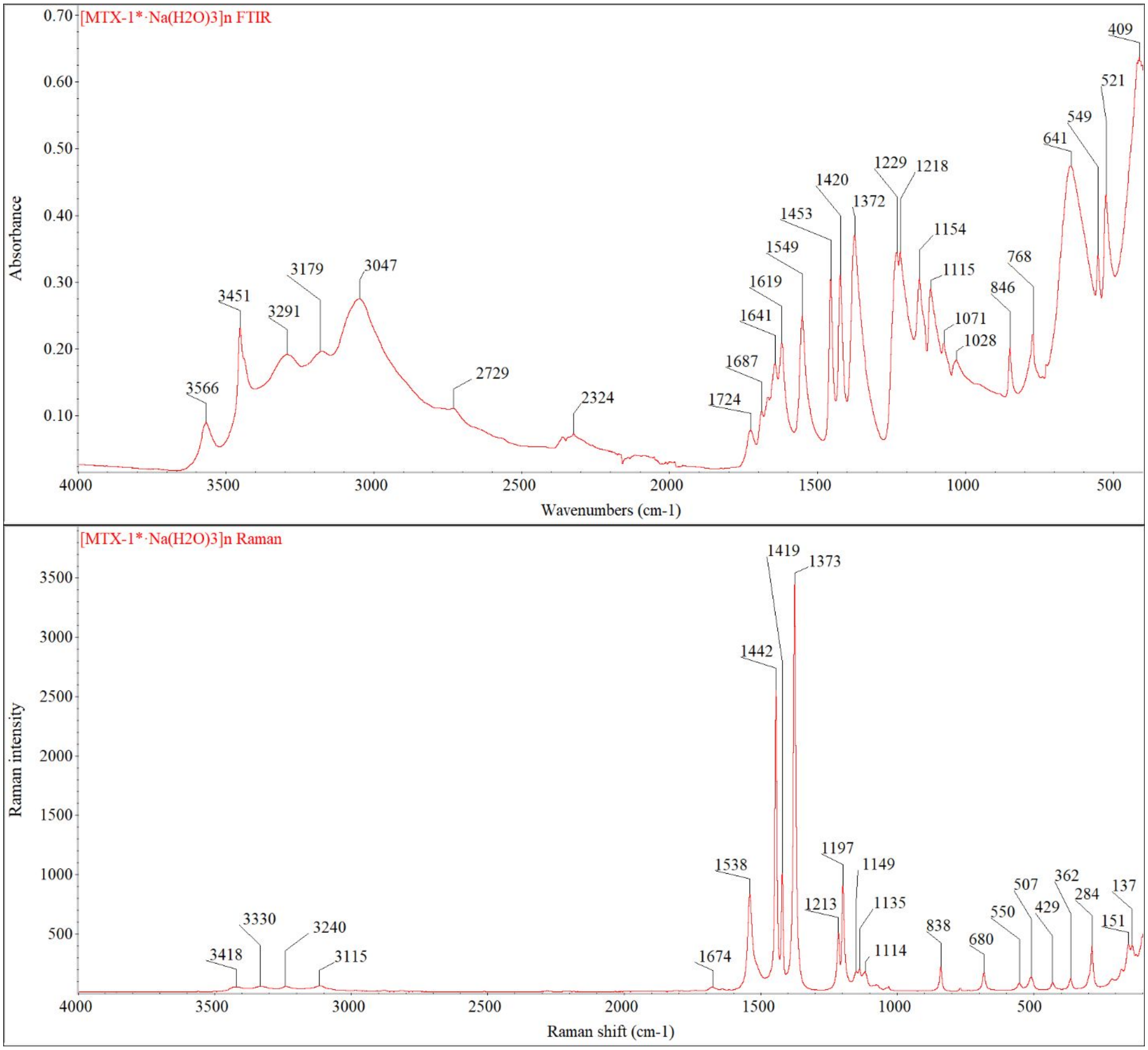

Figure S3. FTIR (top) and Raman (bottom) spectra of **[MTX-1*·Na(H_2O)$_3$]$_n$.**

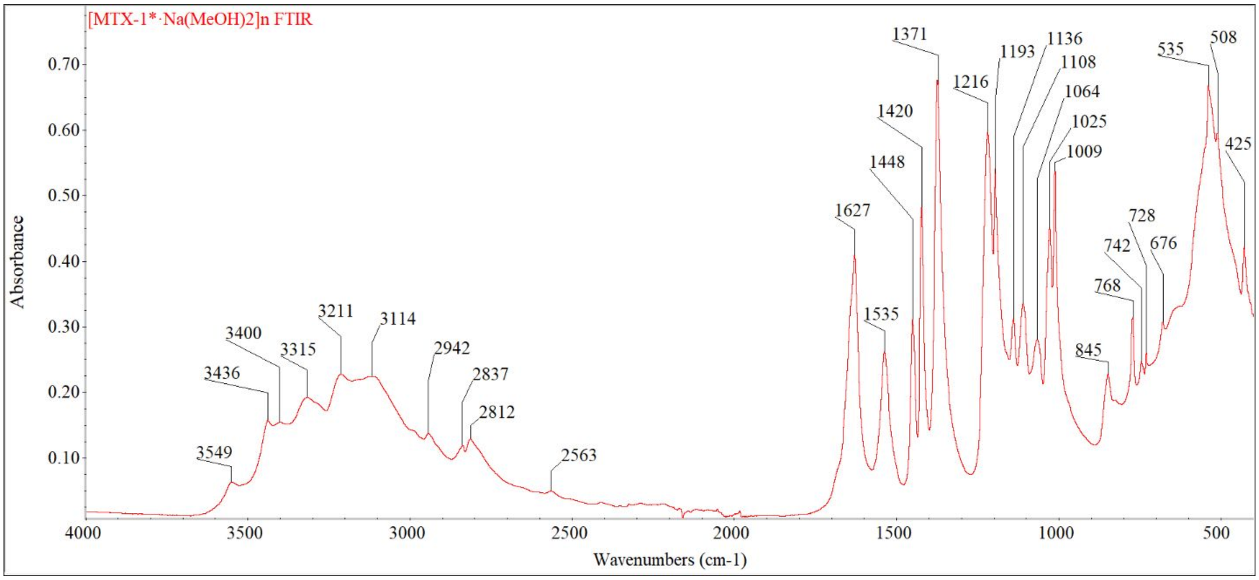

Figure S4. FTIR spectra of **[MTX-1*·Na(MeOH)$_2$]$_n$.**

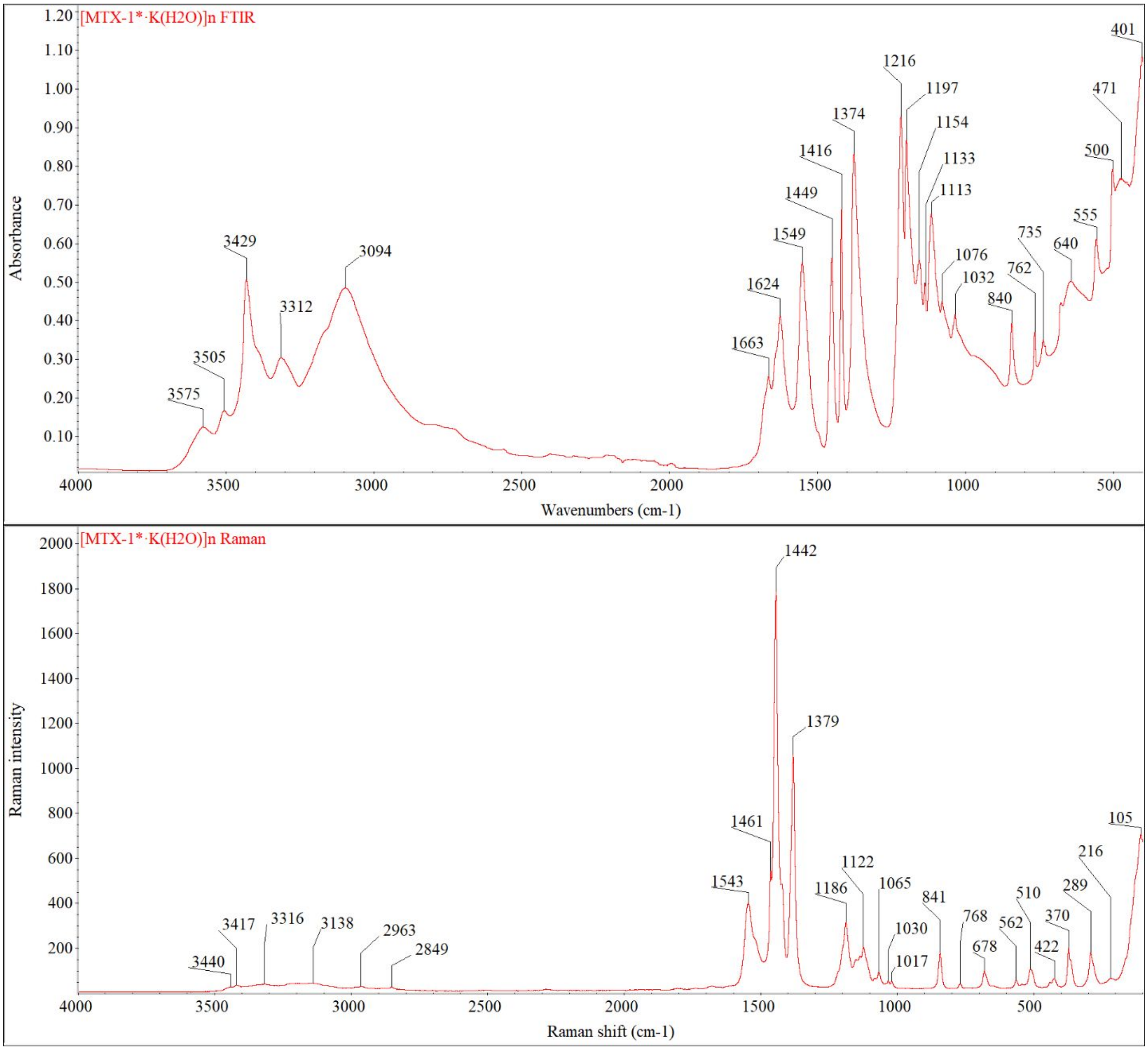

Figure S5. FTIR (top) and Raman (bottom) spectra of **[MTX-1*·K(H$_2$O)]$_n$.**

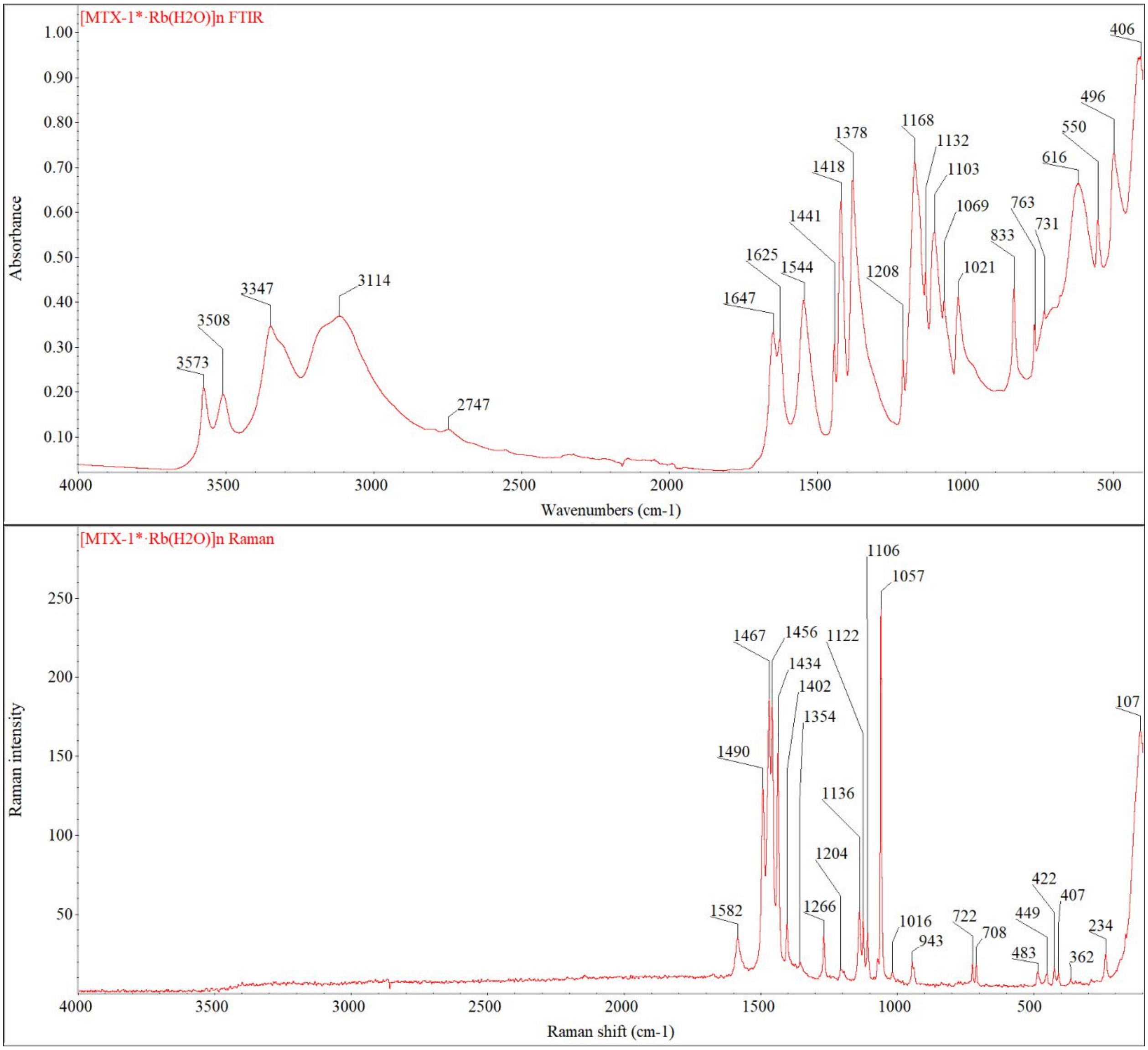

Figure S6. FTIR (top) and Raman (bottom) spectra of **[MTX-1*·Rb(H_2O)]$_n$.**

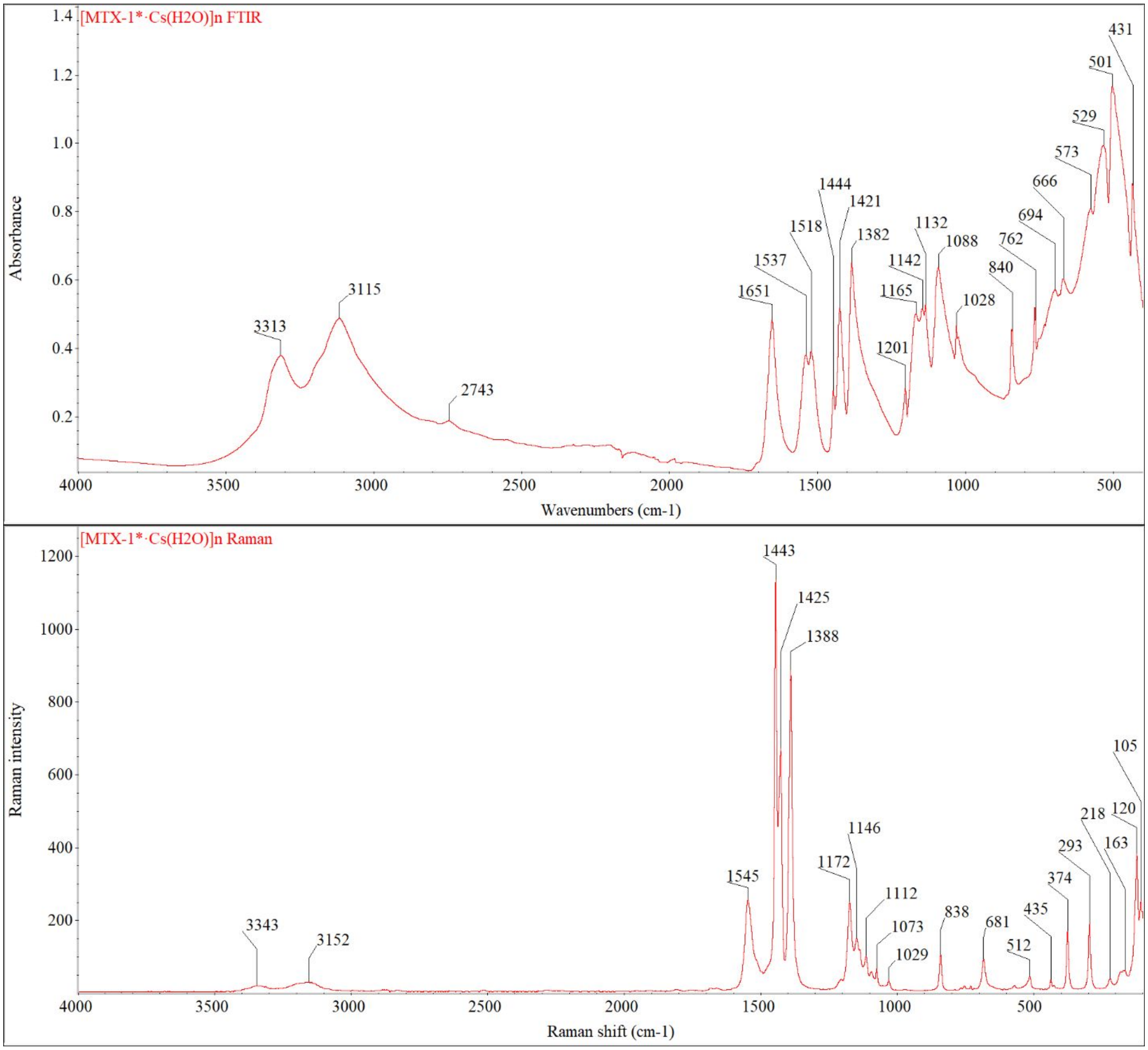

Figure S7. FTIR (top) and Raman (bottom) spectra of **$[MTX\text{-}1^{*}\cdot Cs(H_2O)]_n$.**

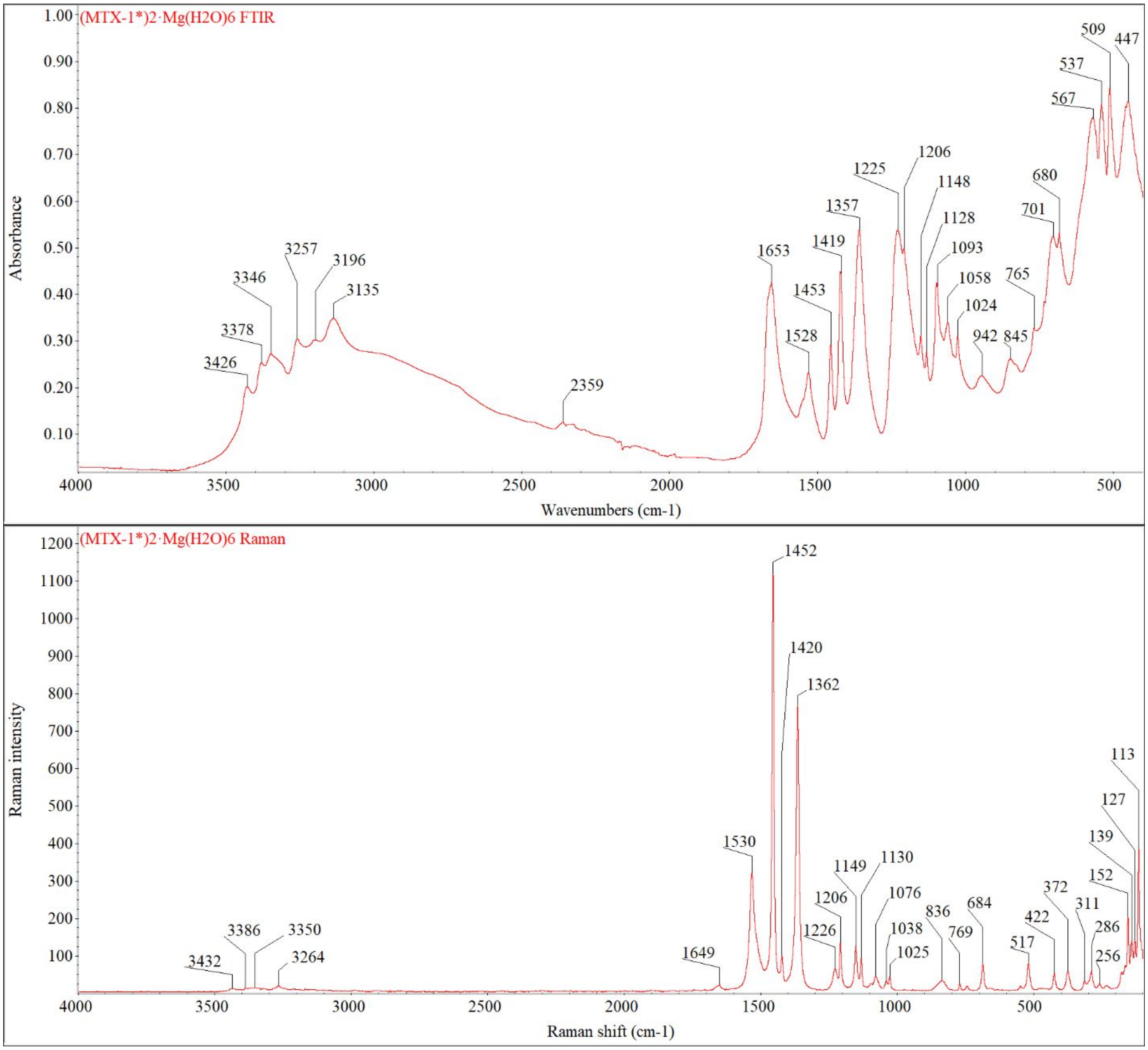

Figure S8. FTIR (top) and Raman (bottom) spectra of **$(MTX\text{-}1^{*})_2\cdot Mg(H_2O)_6$.**

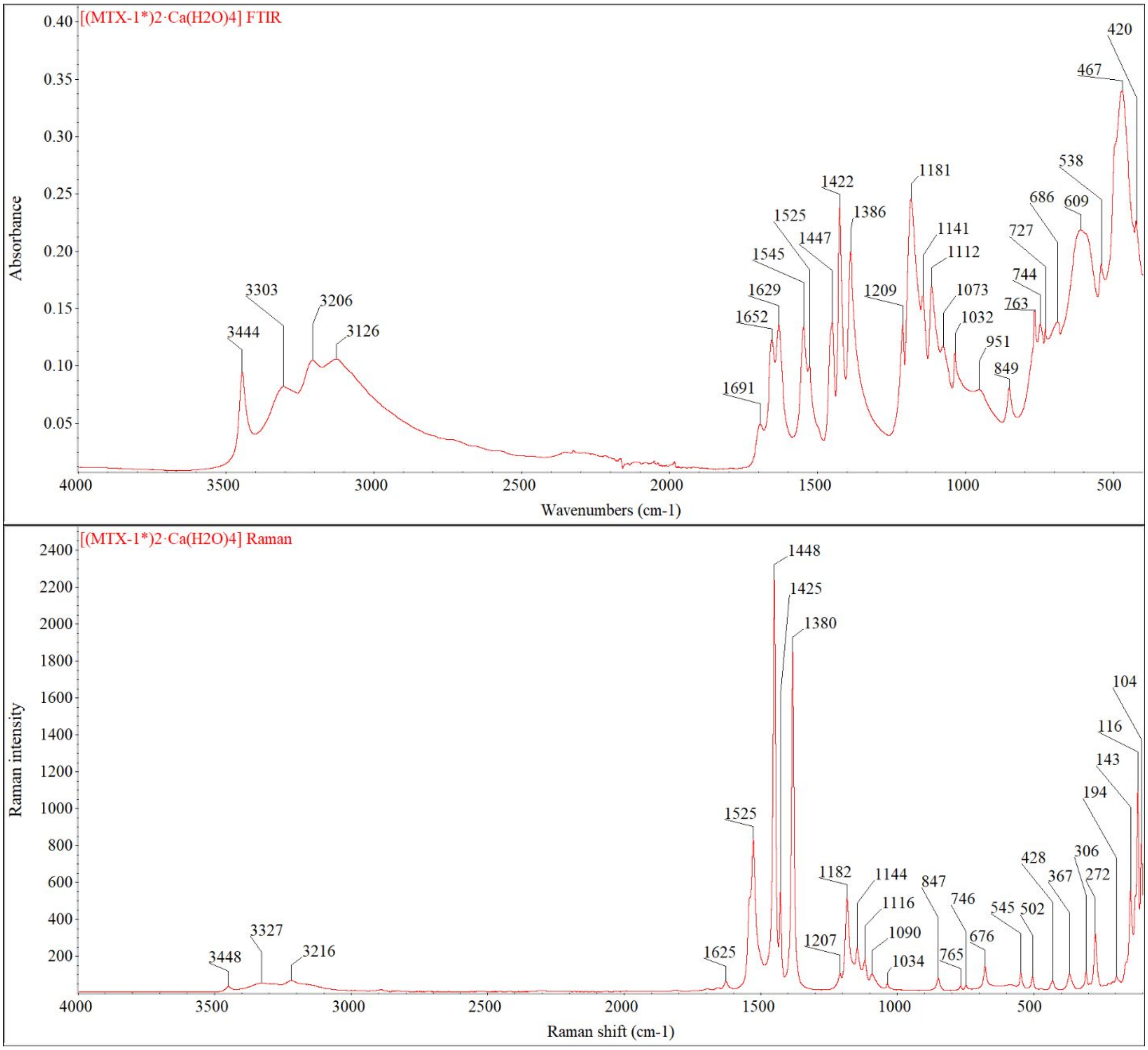

Figure S9. FTIR (top) and Raman (bottom) spectra of **[(MTX-1[*])$_2$·Ca(H$_2$O)$_4$].**

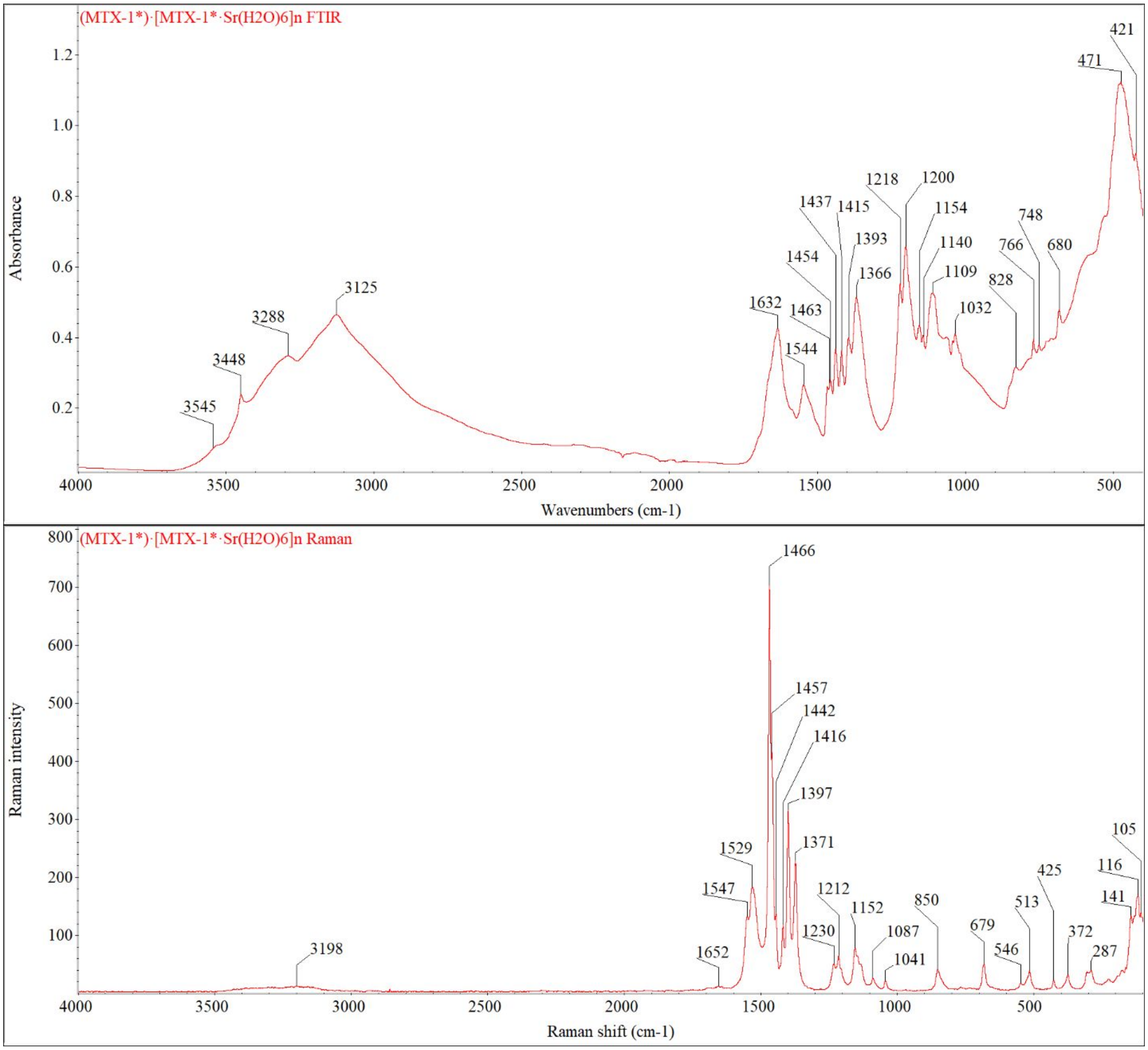

Figure S10. FTIR (top) and Raman (bottom) spectra of **(MTX-1*)·[MTX-1*·Sr(H_2O)$_6$]$_n$**.

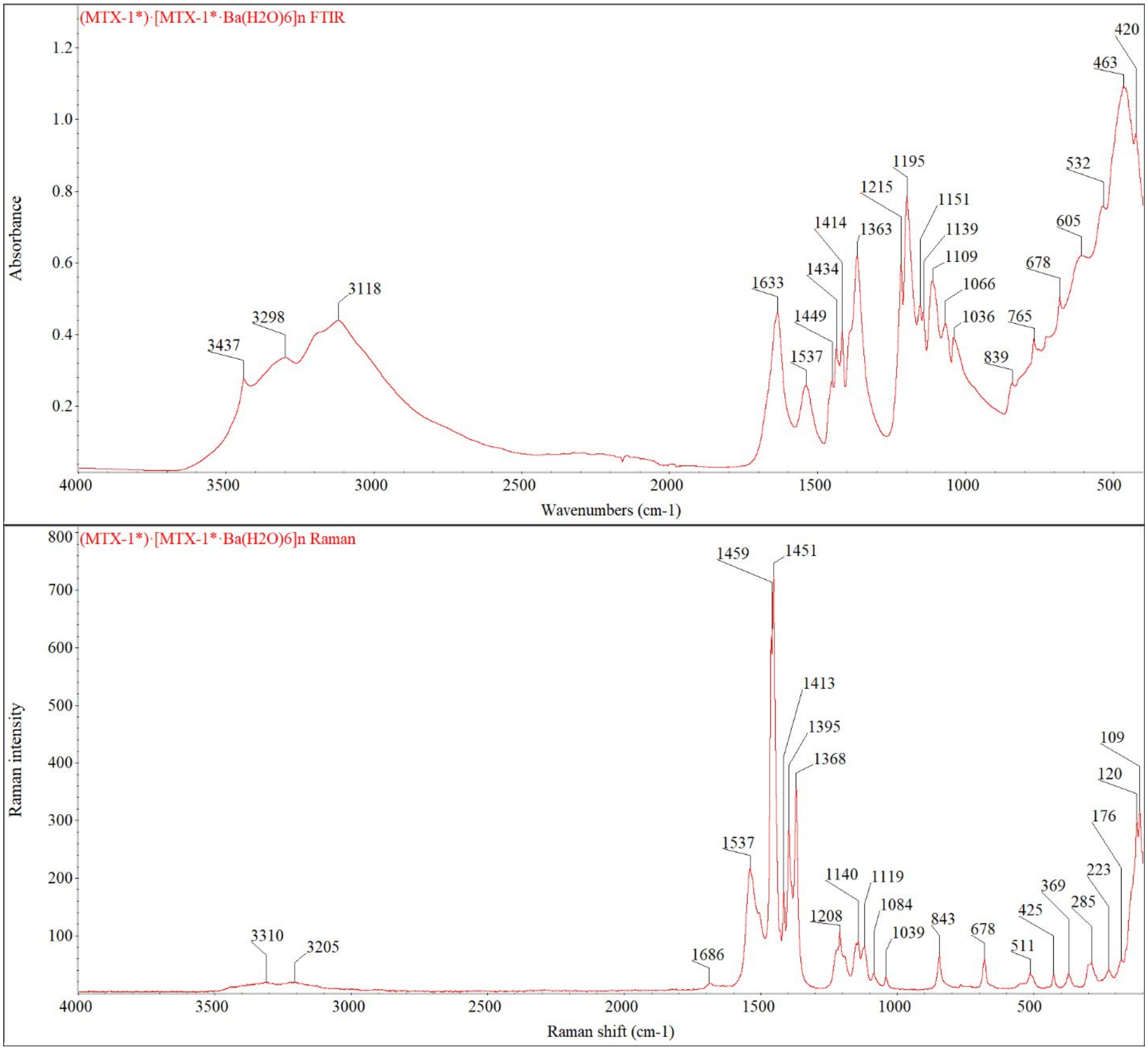

Figure S11. FTIR (top) and Raman (bottom) spectra of **(MTX-1*)·[MTX-1*·Ba(H$_2$O)$_6$]$_n$**.

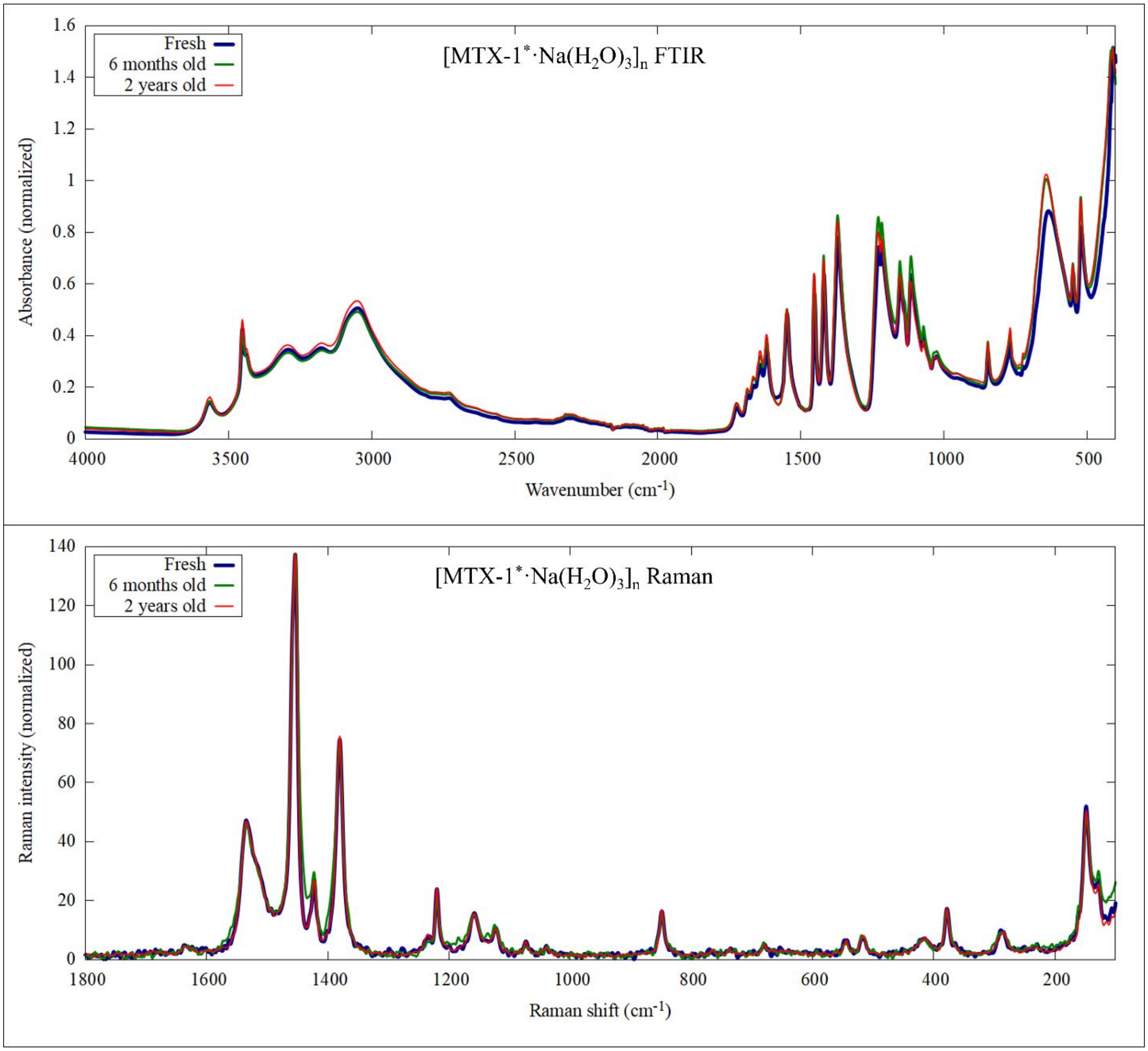

Figure S12. FTIR (top) and Raman (bottom) spectra of **[MTX-1*·Na(H_2O)$_3$]$_n$** samples: fresh (blue), aged for 6 months (green), and aged for 2 years (red) under ambient conditions. The spectra are normalized to demonstrate structural stability.

Long-term stability data

Table S1. Comparison of elemental analysis and decomposition temperatures for fresh and aged samples of **[MTX-1*·Na(H_2O)$_3$]$_n$**.

Sample age	C [%]	H [%]	N [%]	T_{dec} (onset) [°C]
Calculated	10.39	4.36	54.54	-
Fresh	10.42	4.27	55.33	248
6 months	10.47	4.22	54.47	249
2 years	10.46	4.28	55.32	249

Sensitivity data

Table S2. Raw friction and impact sensitivity data of dehydrated **MTX-1** salts. Friction force levels, number of initiations, weight of used hammer and impact energy levels.

**Compound**	**Friction force F [N]**	**No. of initiations**	**Hammer weight [kg]**	**Impact energy E [J]**	**No. of initiations**
**Na MTX-1**	288	2/15	1	3.1	3/15
	324	6/15		5.0	6/15
	360	7/15		6.0	7/15
	376	9/15		7.1	11/15
	408	13/15		9.0	12/15
**K MTX-1**	216	1/15	0.5	1.6	2/15
	252	3/15		2.0	4/15
	288	5/15		2.5	7/15
	324	6/15		4.0	13/15
	360	8/15		5.0	13/15
**Rb MTX-1**	180	3/15	0.5	1.5	2/15
	216	5/15		2.0	5/15
	252	6/15		2.5	9/15
	288	7/15		4.0	11/15
	324	9/15		5.0	14/15
	360	11/15			
**Cs MTX-1**	120	2/15	0.5	0.8	1/15
	160	4/15		1.0	3/15
	180	8/15		1.6	6/15
	240	10/15		2.5	10/15
	288	13/15		3.2	13/15

**Sr (MTX-1)$_2$**	252	3/15	1	1.5	2/15
	324	7/15		2.0	5/15
	360	8/15		3.6	9/15
	396	8/15		5.0	10/15
	456	10/15		8.0	14/15
				10	14/15
**Ba (MTX-1)$_2$**	240	4/15	1	3.0	2/15
	252	6/15		5.0	4/15
	288	7/15		5.6	7/15
	324	8/15		6.3	9/15
	360	12/15		10	13/15

Table S3. Friction and impact sensitivity curves parameters, mean and standard deviation, of dehydrated MTX-1 salts.

**Compound**	**Friction sensitivity curve parameters**		**Impact sensitivity curve parameters**	
	**Mean μ**	**Standard deviation σ**	**Mean μ**	**Standard deviation σ**
**Na MTX-1**	5.87	0.197	1.71	0.604
**K MTX-1**	5.85	0.350	0.971	0.477
**Rb MTX-1**	5.64	0.512	0.915	0.526
**Cs MTX-1**	5.25	0.404	0.583	0.591
**Sr (MTX-1)$_2$**	5.67	0.342	1.78	0.514
**Ba (MTX-1)$_2$**	5.89	0.496	1.13	0.750

Detonation test

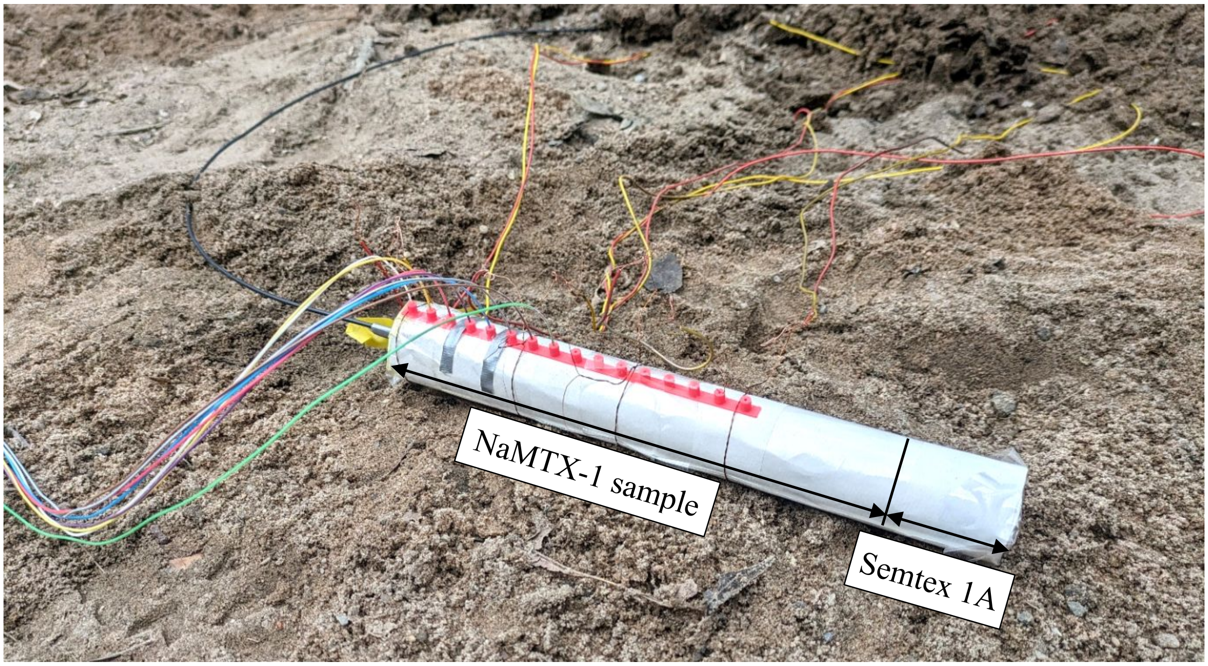

Figure S13. Detonation test of the anhydrous sodium salt MTX-1 (NaMTX-1). Photo of the setup before the test with ionization probes (wires), discrete optic fibers (colored optic fibers), and continuous fiber optic probe (black optic fiber), without the detonator. The sample charge and booster placement are indicated.

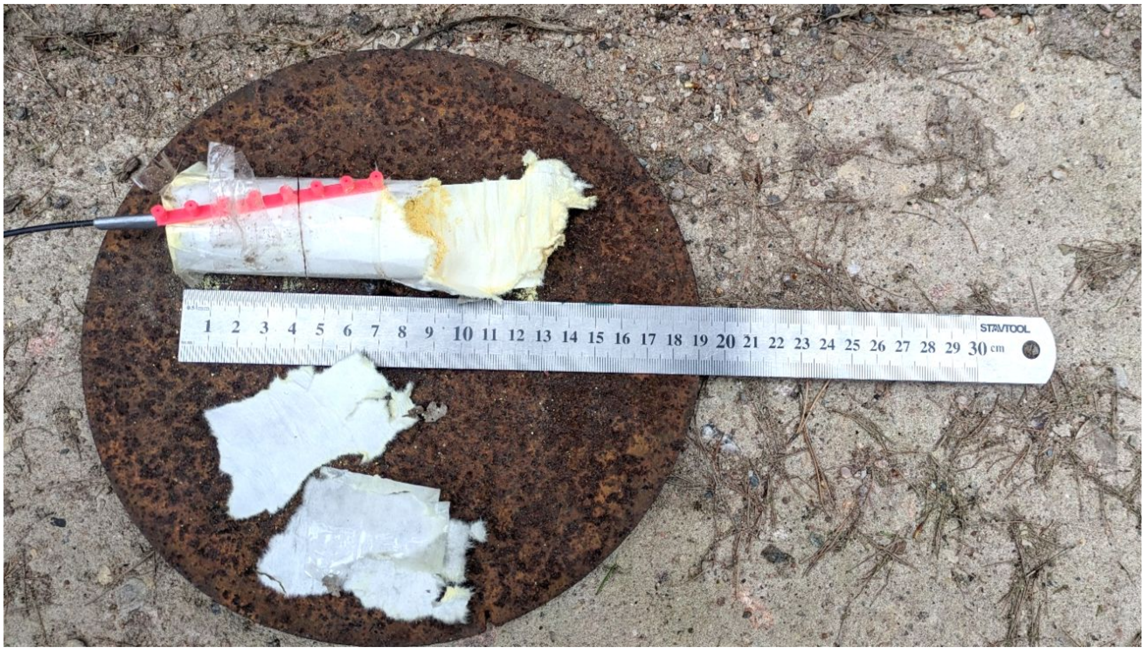

Figure S14. Setup of the detonation test after the test. The anhydrous sodium salt MTX-1 was just partially scattered by the booster, with no observable stable detonation.

X-ray data

Table S4. Comparison of main interatomic distances [Å] and bond angles [°] in **Li-Ba** complexes.

**Coordinated molecules of MTX-1***											
**Com-plex**	**N1-N2**	**N2-N3**	**N3-N4**	**N5-N6**	**N6-N7**	**C1-N1**	**C1-N4**	**C1-N5**	**C2-N7**	**C2-N8**	**C2-N9**
**Li**	1.3446(2)	1.3080(3)	1.3424(2)	1.2777(2)	1.3303(2)	1.3422(2)	1.3375(2)	1.3961(2)	1.3608(3)	1.3310(3)	1.3314(3)
**Na** $_{H_2O}$	1.347(1)	1.3068(1)	1.351(1)	1.291(1)	1.323(1)	1.339(1)	1.340(1)	1.394(1)	1.367(1)	1.3310(3)	1.319(2)
**Na** $_{MeOH}$	1.345(1)	1.316(1)	1.339(1)	1.278(1)	1.333(1)	1.332(1)	1.338(1)	1.404(1)	1.366(1)	1.331(1)	1.327(1)
**K**	1.3437(6)	1.3126(6)	1.3430(6)	1.2789(5)	1.3308(4)	1.3328(5)	1.3412(5)	1.3978(5)	1.3610(5)	1.3337(6)	1.3305(5)
**Rb**	1.3409(8)	1.3058(8)	1.3437(7)	1.2694(6)	1.3392(7)	1.3348(7)	1.3358(7)	1.3984(7)	1.3549(7)	1.3235(7)	1.3444(8)
**Cs**	1.347(2)	1.313(2)	1.343(2)	1.268(1)	1.342(1)	1.336(1)	1.337(1)	1.401(1)	1.360(2)	1.331(2)	1.335(2)
**Ca**	1.348(1)	1.318(1)	1.340(1)	1.275(1)	1.345(1)	1.333(1)	1.339(1)	1.403(1)	1.365(1)	1.324(1)	1.330(1)
**Sr**	1.347(3)	1.315(3)	1.343(3)	1.277(3)	1.335(3)	1.338(3)	1.334(3)	1.394(3)	1.369(3)	1.325(3)	1.322(3)
**Ba**	1.349(2)	1.319(2)	1.347(2)	1.275(2)	1.340(2)	1.336(2)	1.337(2)	1.397(2)	1.368(2)	1.328(3)	1.325(2)
**Range**	1.3409(8)-1.349(2)	1.3058(8)-1.319(2)	1.339(1)-1.351(1)	1.268(1)-1.291(1)	1.323(1)-1.345(1)	1.332(1)-1.3422(2)	1.334(3)-1.3412(5)	1.394(1)-1.404(1)	1.3549(7)-1.369(3)	1.323(3)-1.3337(6)	1.319(2)-1.3444(8)
**Bond length**	**N…Li**	**N…Na**	**N…Na***	**N…K**	**N…Rb**	**N…Cs**	**N…Ca**	**N…Sr**	**N…Ba**		
	2.0300(6)-2.0538(6)	2.560(1)	2.4098(9)	2.8256(5)	2.9824(6) 3.3631(6)	3.191(1) 3.239(1)	2.4801(8)	2.766(2)	2.933(2)		

Non-coordinated molecules of MTX-1*											
**Complex**	**N1-N2**	**N2-N3**	**N3-N4**	**N5-N6**	**N6-N7**	**C1-N1**	**C1-N4**	**C1-N5**	**C2-N7**	**C2-N8**	**C2-N9**
**Mg**	1.350(1)	1.309(1)	1.354(1)	1.285(1)	1.323(1)	1.331(1)	1.344(1)	1.399(1)	1.374(1)	1.321(1)	1.327(1)
**Complex**	**N10-N11**	**N11-N12**	**N12-N13**	**N14-N15**	**N15-N16**	**C3-N10**	**C3-N13**	**C3-N14**	**C4-N16**	**C4-N17**	**C4-N18**
**Sr**	1.338(3)	1.314(3)	1.345(3)	1.282(3)	1.335(3)	1.329(3)	1.341(3)	1.396(3)	1.357(3)	1.331(3)	1.328(3)
**Ba**	1.336(2)	1.320(2)	1.348(2)	1.298(2)	1.327(2)	1.342(2)	1.336(2)	1.399(2)	1.368(3)	1.340(3)	1.320(3)
**Range**	1.336(2)-1.350(1)	1.309(1)-1.320(2)	1.345(3)-1.354(1)	1.282(3)-1.298(2)	1.323(1)-1.335(3)	1.329(3)-1.342(2)	1.336(2)-1.344(1)	1.396(3)-1.399(2)	1.357(3)-1.374(1)	1.321(1)-1.340(3)	1.320(3)-1.328(3)
**The angle between planes of the head and the tail in MTX-1 (free is non-coordinated molecule)**											
**Li**	**Na H_2O**	**Na MeOH**	**K**	**Rb**	**Cs**	**6 Mg (free)**	**7 Ca**	**8 Sr**	**9 Ba**	**8 Sr (free)**	**9 Ba (free)**
8.02	19.70	12.84	9.49	1.57	20.13	14.47	11.50	5.22	7.27	17.01	17.58

Table S5. Selected crystallographic data of Li-Ba complexes.

Compound	**[MTX-1*·Li(H2O)2]2**	**[MTX-1*·Na(H2O)3]n**	**[MTX-1* ·Na(MeOH)2]n**	**[MTX-1*·K(H2O)]n**
CCDC deposition number	2482912	2482907	2482905	2482914
Empirical formula	$(C_2N_4H_9)_2{\cdot}Li_2(H_2O)_4$	$C_2H_4N_9{\cdot}Na(H_2O)_3$	$C_2H_4N_9{\cdot}Na(MeOH)_2$	$C_2H_4N_9{\cdot}K(H_2O)$, H_2O
Formula weight	394.23	231.18	241.22	229.27
Temperature/K	150(2)	150(2)	150(2)	150(2)
Crystal system	Triclinic	Monoclinic	Monoclinic	Orthorhombic
Space group	*P-1*	$P2_1/c$	$P2_1/n$	$Pna2_1$
a/Å	6.5951(2)	3.63570(10)	9.8812(2)	16.1068(3)
b/Å	7.8139(2)	15.4748(3)	8.6062(2)	15.7192(3)
c/Å	8.7534(2)	16.7736(3)	12.9684(3)	3.59570(10)
α/°	94.6390(10)	90	90	90
β/°	100.7600(10)	93.6670(10)	96.7930(10)	90
γ/°	111.6620(10)	90	90	90
Volume/Å^3	406.284(19)	941.78(4)	1095.09(4)	910.38(4)
Z	1	4	4	4
ρ_{calc} g/cm^3	1.611	1.630	1.463	1.673
μ/mm^{-1}	0.134	0.178	0.150	0.580
F(000)	204	480	504	472
Crystal size/mm	0.472 x 0.233 x 0.190	0.507 x 0.296 x 0.210	0.594 x 0.348 x 0.238	0.346 x 0.218 x 0.124
Radiation	Mo Kα	Mo Kα	Mo Kα	Mo Kα
Θ range for data collection/°	2.401 to 52.217	2.433 to 27.527	3.149 to 28.289	1.810 to 52.155
Index ranges	-14<=h<=14, -17<=k<=17, -19<=l<=19	-4<=h<=4, -20<=k<=20, -21<=l<=21	-13<=h<=13, -11<=k<=11, -17<=l<=17	-35<=h<=35, -34<=k<=34, -7<=l<=7
Reflections collected	191034	77607	26214	117994
Independent reflections	9207 [R(int) = 0.0230]	2163 [R(int) = 0.0253]	2689 [R(int) = 0.0321]	10049 [R(int) = 0.0321]
Data/restraints/parameters	9207 / 0 / 159	2163 / 0 / 160	2689 / 108 / 165	10049 / 1 / 159
Goodness-of-fit on F^2	1.127	1.042	1.093	1.143
Final R indexes [I≥2σ(I)]	R1 = 0.0255, wR2 = 0.0799	R1 = 0.0306, wR2 = 0.0791	R1 = 0.0284, wR2 = 0.0799	R1 = 0.0238, wR2 = 0.0671
Final R indexes [all data]	R1 = 0.0266, wR2 = 0.0810	R1 = 0.0307, wR2 = 0.0792	R1 = 0.0302, wR2 = 0.0818	R1 = 0.0248, wR2 = 0.0706
Largest diff. peak/hole / e Å^{-3}	0.521 and -0.273	0.545 and -0.314	0.283 and -0.193	0.709 and -0.306

Compound	**[MTX-1*·Rb(H_2O)]$_n$**	**[MTX-1*·Cs(H_2O)]$_n$**	**(MTX-1*)$_2$ ·Mg(H_2O)$_6$**	**[(MTX-1*)$_2$ ·Ca(H_2O)$_4$]**
CCDC deposition number	2482913	2482910	2482911	2482906
Empirical formula	$C_2H_4N_9$·Rb(H_2O), H_2O	$C_2H_4N_9$·Cs(H_2O)	Mg(H_2O)$_6$, ($C_2H_4N_9$)$_2$	($C_2H_4N_9$)$_2$·Ca(H_2O)$_4$
Formula weight	275.64	305.07	440.69	420.43
Temperature/K	150(2)	150(2)	150(2)	150(2)
Crystal system	Monoclinic	Orthorhombic	Triclinic	Orthorhombic
Space group	*P2₁/c*	*P2₁2₁2₁*	*P-1*	*Pccn*
a/Å	9.4600(4)	6.8379(2)	7.0853(2)	6.76810(10)
b/Å	6.8253(3)	9.2086(2)	8.2418(2)	14.2113(3)
c/Å	15.0078(7)	13.3913(3)	9.0720(2)	16.9093(4)
α/°	90	90	99.0820(10)	90
β/°	107.776(2)	90	109.4240(10)	90
γ/°	90	90	112.6920(10)	90
Volume/Å^3	922.75(7)	843.22(4)	435.299(19)	1626.40(6)
Z	4	4	1	4
ρ_{calc} g/cm^3	1.984	2.403	1.681	1.717
μ/mm^{-1}	5.362	4.372	0.177	0.450
F(000)	544	576	230	872
Crystal size/mm	0.533 x 0.360 x 0.264	0.401 x 0.270 x 0.206	0.320 x 0.090 x 0.061	0.396 x 0.174 x 0.118
Radiation	Mo Kα	Mo Kα	Mo Kα	Mo Kα
Θ range for data collection/°	2.851 to 52.558	3.345 to 52.234	2.521 to 28.285	2.409 to 28.295
Index ranges	-21<=h<=21, -15<=k<=15, -33<=l<=33	-15<=h<=15, -20<=k<=20, -29<=l<=29	-9<=h<=9, -10<=k<=10, -12<=l<=12	-9<=h<=8, -18<=k<=18, -22<=l<=22
Reflections collected	296550	142181	16469	34295
Independent reflections	10613 [R(int) = 0.0610]	9679 [R(int) = 0.0416]	2148 [R(int) = 0.0385]	1985 [R(int) = 0.0302]
Data/restraints/parameters	10613 / 0 / 139	9679 / 2 / 125	2148 / 0 / 164	1985 / 0 / 147
Goodness-of-fit on F^2	1.068	1.204	1.063	1.059
Final R indexes [I≥2σ(I)]	R1 = 0.0274, wR2 = 0.0677	R1 = 0.0205, wR2 = 0.0548	R1 = 0.0271, wR2 = 0.0695	R1 = 0.0229, wR2 = 0.0616
Final R indexes [all data]	R1 = 0.0317, wR2 = 0.0696	R1 = 0.0205, wR2 = 0.0548	R1 = 0.0307, wR2 = 0.0724	R1 = 0.0237, wR2 = 0.0623
Largest diff. peak/hole / e Å^{-3}	1.399 and -2.212	1.163 and -2.149	0.282 and -0.228	0.289 and -0.251

Compound	**(MTX-1*)·[MTX-1*·Sr(H$_2$O)$_6$]$_n$**	**(MTX-1*)·[MTX-1*·Ba(H$_2$O)$_6$]$_n$**
CCDC deposition number	2482908	2482909
Empirical formula	$C_2H_4N_9 \cdot Sr(H_2O)_5$, $C_2H_4N_9$, $3 \cdot H_2O$	$C_2H_4N_9 \cdot Ba(H_2O)_5$, $C_2H_4N_9$, $3 \cdot H_2O$
Formula weight	540.03	589.75
Temperature/K	150(2)	150(2)
Crystal system	Monoclinic	Monoclinic
Space group	*C2/c*	*C2/c*
a/Å	34.883(2)	35.0054(16)
b/Å	6.7651(5)	6.8227(3)
c/Å	17.0598(12)	17.2940(7)
α/°	90	90
β/°	98.009(3)	97.873(3)
γ/°	90	90
Volume/Å^3	3986.6(5)	4091.4(3)
Z	8	8
ρ_{calc} g/cm^3	1.800	1.915
μ/mm^{-1}	2.784	2.019
F(000)	2208	2352
Crystal size/mm	0.338 x 0.131 x 0.079	0.227 x 0.087 x 0.040
Radiation	Mo Kα	Mo Kα
Θ range for data collection/°	2.358 to 28.316	2.378 to 28.380
Index ranges	-46<=h<=46, -9<=k<=9, -22<=l<=22	-46<=h<=46, -9<=k<=9, -23<=l<=23
Reflections collected	57976	125113
Independent reflections	4929 [R(int) = 0.0932]	5090 [R(int) = 0.0649]
Data/restraints/parameters	4929 / 5 / 353	5090 / 0 / 353
Goodness-of-fit on F^2	1.090	1.030
Final R indexes [I≥2σ(I)]	R1 = 0.0325, wR2 = 0.0779	R1 = 0.0191, wR2 = 0.0418
Final R indexes [all data]	R1 = 0.0509, wR2 = 0.0853	R1 = 0.0240, wR2 = 0.0441
Largest diff. peak/hole / e Å^{-3}	0.722 and -0.863	0.778 and -0.581

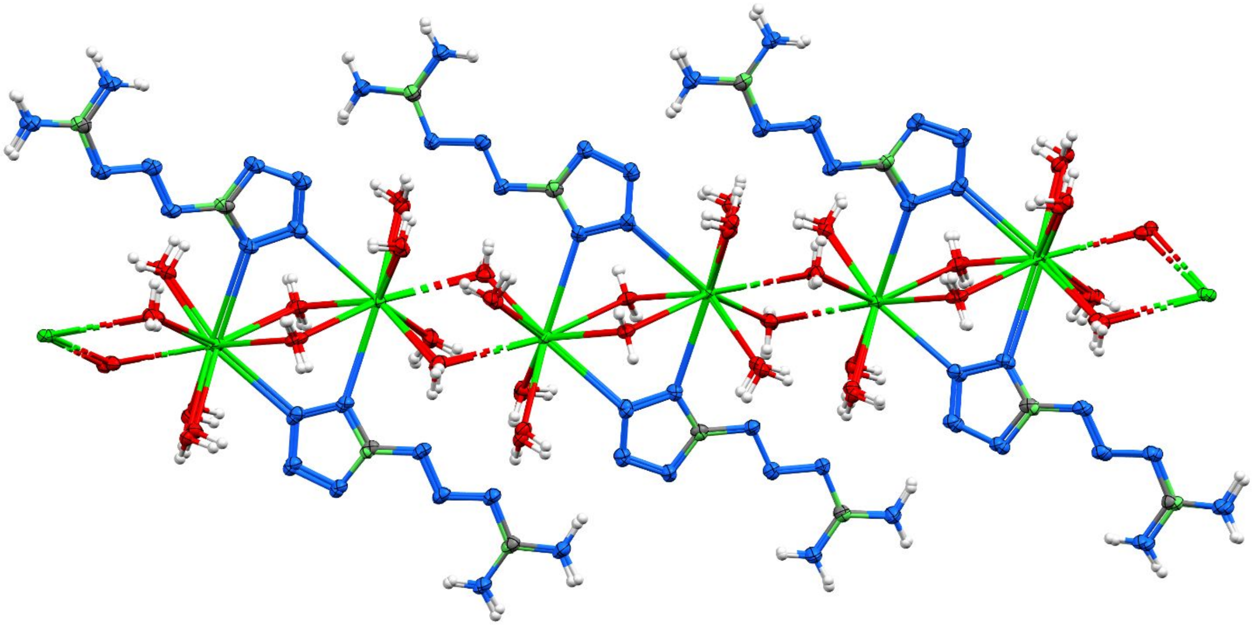

Figure S15. Overlapping structural motifs of **(MTX-1*)·[MTX-1*·Sr(H$_2$O)$_6$]$_n$** (grey carbons) and **(MTX-1*)·[MTX-1*·Ba(H$_2$O)$_6$]$_n$** (green carbons). ORTEP diagram drawn at 50% probability level; non-coordinated water molecules are omitted for clarity.

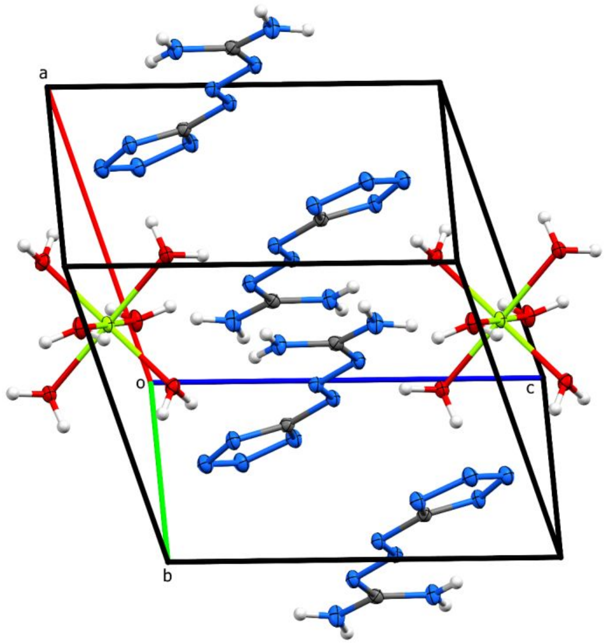

Figure S16. Fragment of crystal packing of **(MTX-1*)$_2$·Mg(H$_2$O)$_6$**. An ORTEP diagram, 50% probability level.

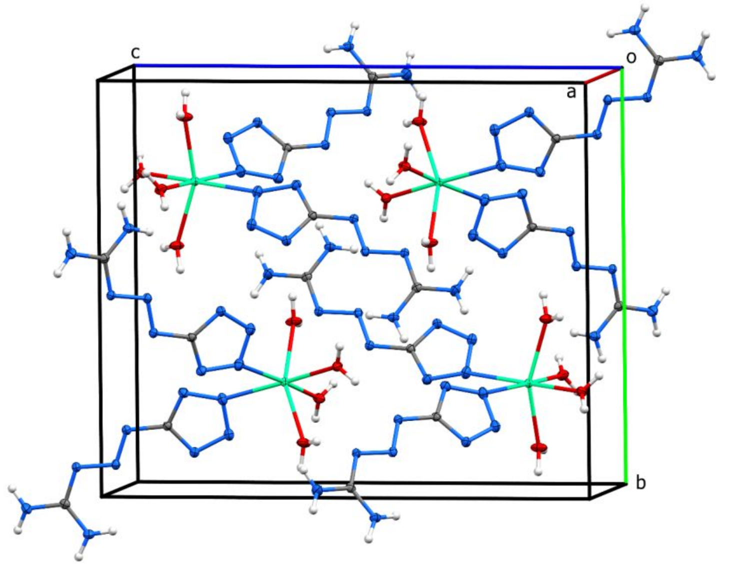

Figure S17. Fragment of crystal packing of **[(MTX-1*)$_2$·Ca(H$_2$O)$_4$]**. An ORTEP diagram, 50% probability level.

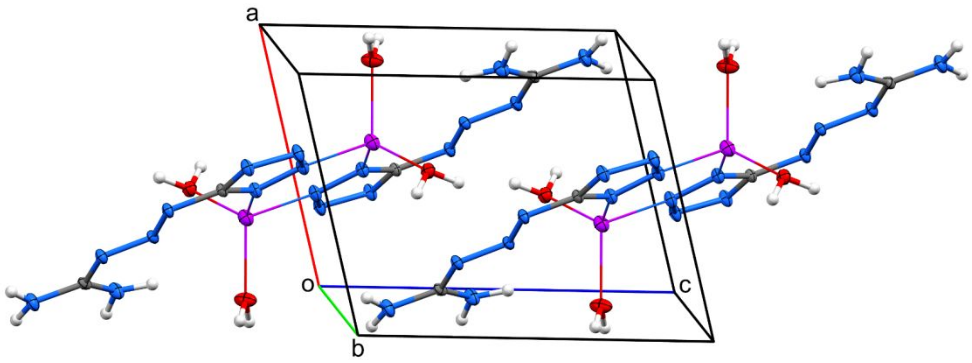

Figure S18. Fragment of crystal packing of **[MTX-1*·Li(H$_2$O)$_2$]$_2$**. An ORTEP diagram, 50% probability level.

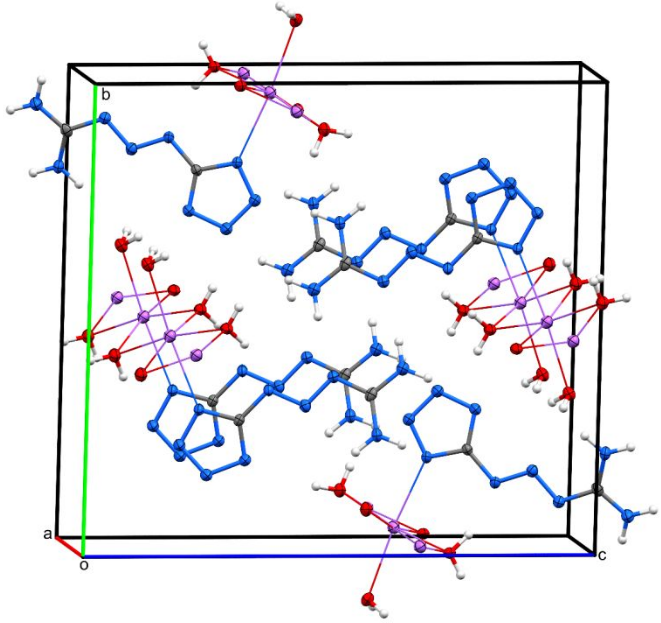

Figure S19. Fragment of crystal packing of **[MTX-1*·Na(H_2O)$_3$]$_n$**. An ORTEP diagram, 50% probability level.

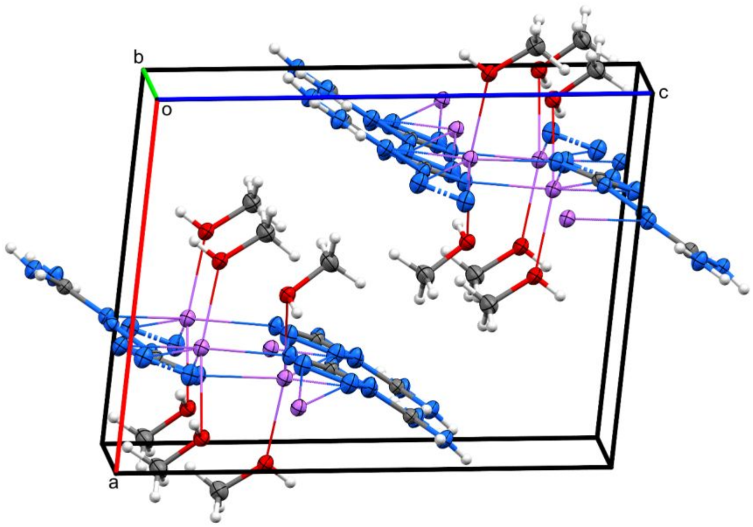

Figure S20. Fragment of crystal packing of **[MTX-1*·Na(MeOH)$_2$]$_n$**. An ORTEP diagram, 50% probability level.

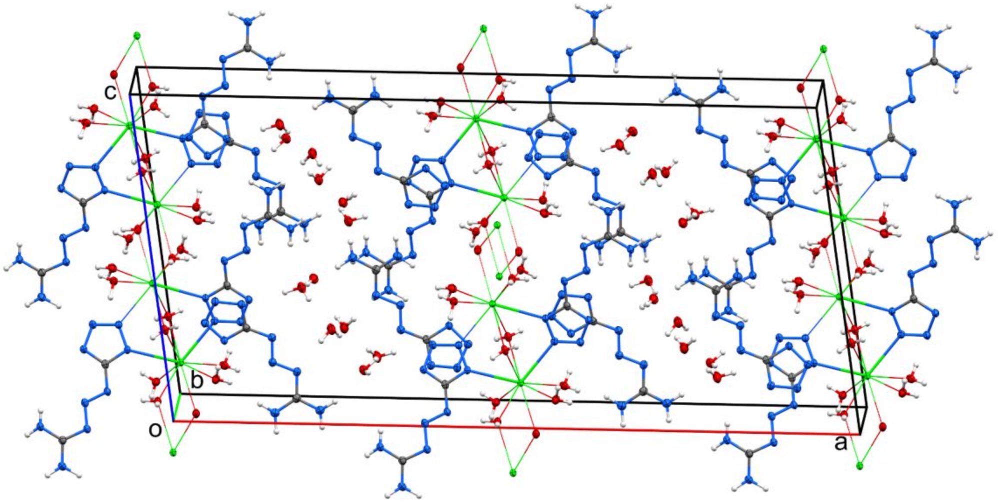

Figure S21. Fragment of crystal packing of **(MTX-1*)·[MTX-1*·Sr(H_2O)$_6$]$_n$**, **(MTX-1*)·[MTX-1*·Ba(H_2O)$_6$]$_n$**. An ORTEP diagram, 50% probability level.

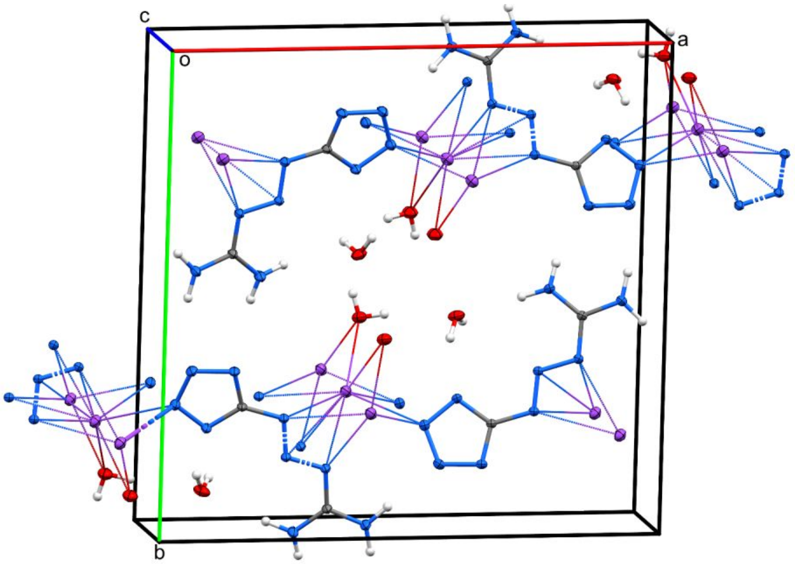

Figure S22. Fragment of crystal packing of **[MTX-1*·K(H_2O)]$_n$**. An ORTEP diagram, 50% probability level.

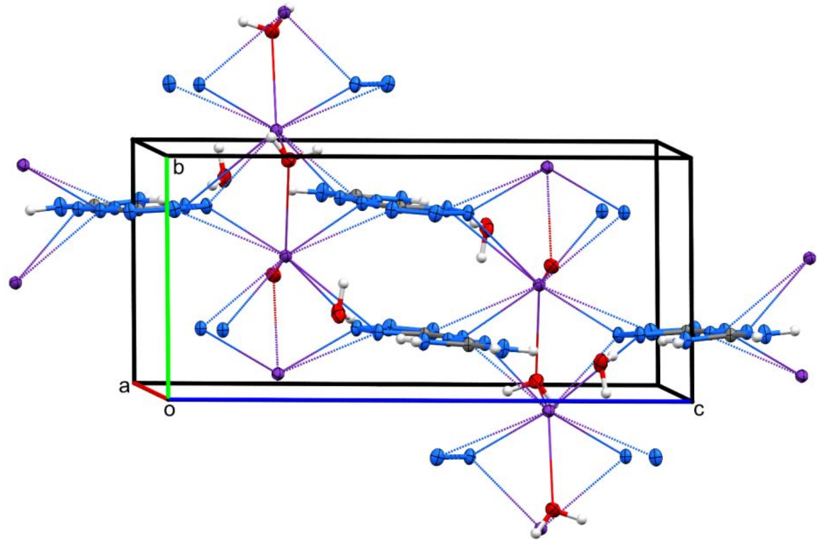

Figure S23. Fragment of crystal packing of **[MTX-1*·Rb(H_2O)]$_n$**. An ORTEP diagram, 50% probability level.

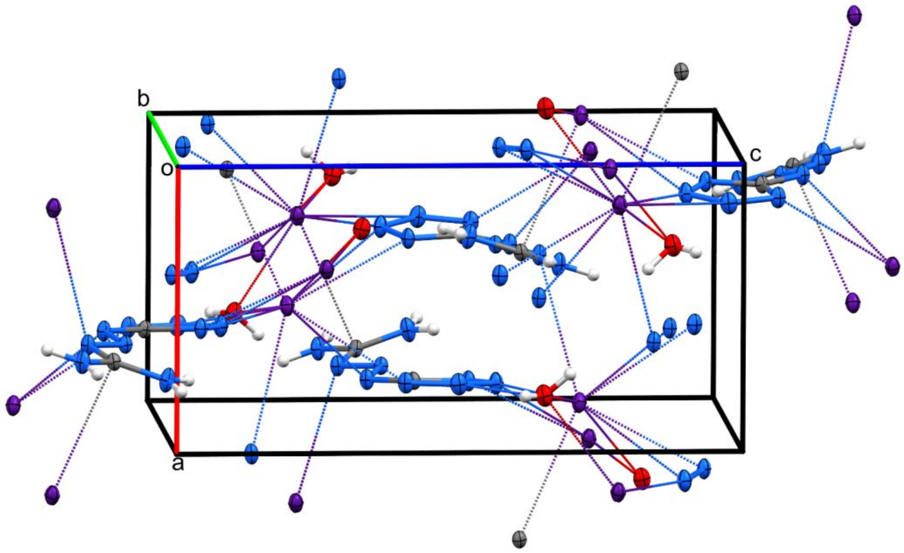

Figure S24. Fragment of crystal packing of **[MTX-1*·Cs(H_2O)]$_n$**. An ORTEP diagram, 50% probability level.

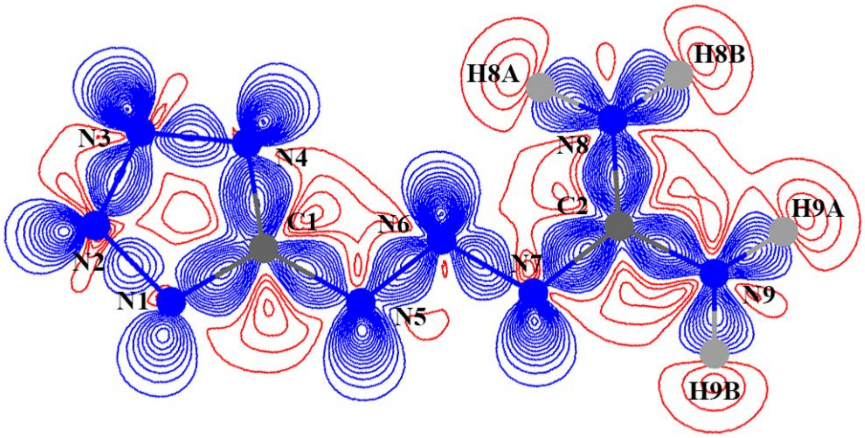

Figure S25. The static deformation electron density distribution in **MTX-1*** anion from **^{ED}[MTX-1*·Li(H_2O)$_2$]$_2$** based on N5-N6-N7 plane. The positive (blue) and negative (red) contours are drawn at intervals 0.05 e/Å^3.

Theoretical data

Table S6. The topological properties of BCPs (3,-1) in small clusters from **Li-Ba** (e/bohr^3), the energies of intermolecular interactions E_{int} (kcal/mol) and corresponding distances (Å).*

Atom 1	Atom 2	Distance	$\rho(r_{cp})$	$\nabla^2\rho(r_{cp})$	$G(r_{cp})$	$V(r_{cp})$	E_{int}
**[MTX-1*·Li(H$_2$O)$_2$]$_2$**							
N26	N56	3.290	0.006	0.023	0.005	-0.003	-0.941
N27	N100	3.410	0.006	0.020	0.004	-0.003	-0.941
C37	N52	3.371	0.007	0.023	0.005	-0.004	-1.255
N28	N97	3.212	0.007	0.025	0.005	-0.004	-1.255
N29	N51	3.222	0.006	0.022	0.004	-0.003	-0.941
N29	N96	3.362	0.006	0.019	0.004	-0.003	-0.941
N30	C59	3.371	0.007	0.023	0.005	-0.004	-1.255
N30	N95	3.362	0.006	0.019	0.004	-0.003	-0.941
N31	N94	3.212	0.007	0.025	0.005	-0.004	-1.255
N34	N48	3.290	0.006	0.023	0.005	-0.003	-0.941
N34	N93	3.410	0.006	0.020	0.004	-0.003	-0.941
**^{ED}[MTX-1*·Li(H$_2$O)$_2$]$_2$**							
C1	N7	3.371	0.048	0.005	0.004	-0.003	-1.054
N3	N9	3.289	0.048	0.006	0.005	-0.004	-1.114
N4	N9	3.410	0.034	0.005	0.004	-0.002	-0.772
N5	N8	3.212	0.042	0.006	0.005	-0.003	-1.042
N6	N6	3.221	0.047	0.005	0.005	-0.003	-1.054
N7	C1	3.371	0.047	0.005	0.004	-0.003	-1.045
N8	N5	3.212	0.042	0.006	0.005	-0.003	-1.039
N9	N3	3.289	0.048	0.006	0.005	-0.004	-1.104
N9	N4	3.410	0.034	0.005	0.004	-0.002	-0.772
**[MTX-1*·Na(H$_2$O)$_3$]$_n$**							
N10	C31	3.491	0.004	0.014	0.003	-0.002	-0.628
N12	N36	3.473	0.004	0.014	0.003	-0.002	-0.628
N13	N59	3.473	0.004	0.014	0.003	-0.002	-0.628
C8	N57	3.491	0.004	0.014	0.003	-0.002	-0.628
N14	N38	3.350	0.005	0.017	0.003	-0.002	-0.628
N15	N61	3.350	0.005	0.017	0.003	-0.002	-0.628
N16	N40	3.355	0.007	0.025	0.005	-0.004	-1.255

N17	N63	3.355	0.007	0.025	0.005	-0.004	-1.255
Atom 1	Atom 2	Distance	$\rho(r_{cp})$	$\nabla^2\rho(r_{cp})$	$G(r_{cp})$	$V(r_{cp})$	E_{int}
**[MTX-1*·Na(H$_2$O)$_3$]$_n$**							
N20	N43	3.636	0.003	0.012	0.002	-0.002	-0.628
N20	N67	3.636	0.003	0.012	0.002	-0.002	-0.628
**[MTX-1*·Na(MeOH)$_2$]$_n$**							
N33	N67	3.383	0.006	0.020	0.004	-0.003	-0.941
N34	N70	3.249	0.007	0.024	0.005	-0.004	-1.255
N40	N64	3.249	0.007	0.024	0.005	-0.004	-1.255
N37	N63	3.383	0.006	0.020	0.005	-0.003	-0.941
**[MTX-1*·K(H$_2$O)]$_n$**							
N17	N37	3.485	0.005	0.017	0.003	-0.003	-0.941
N18	N58	3.360	0.005	0.017	0.003	-0.003	-0.941
N53	N2	3.360	0.005	0.017	0.003	-0.003	-0.941
N19	N1	3.485	0.005	0.017	0.003	-0.003	-0.941
N20	N4	3.596	0.003	0.011	0.002	-0.001	-0.314
N20	N38	3.596	0.003	0.011	0.002	-0.001	-0.314
N22	N6	3.596	0.004	0.013	0.002	-0.002	-0.628
N22	N40	3.596	0.004	0.013	0.002	-0.002	-0.628
C33	N41	3.453	0.004	0.015	0.003	-0.002	-0.628
N23	C16	3.453	0.004	0.015	0.003	-0.002	-0.628
N26	N10	3.596	0.004	0.013	0.002	-0.002	-0.628
N26	N44	3.596	0.004	0.013	0.002	-0.002	-0.628
**[MTX-1*·Rb(H$_2$O)]$_n$**							
N38	N4	3.420	0.005	0.018	0.004	-0.003	-0.941
N38	N23	3.457	0.005	0.016	0.003	-0.002	-0.628
N39	N22	3.422	0.005	0.016	0.003	-0.002	-0.628
N40	N58	3.586	0.004	0.014	0.003	-0.002	-0.628
N41	N1	3.420	0.005	0.016	0.003	-0.003	-0.941
N41	N57	3.457	0.005	0.018	0.004	-0.002	-0.628
N42	N2	3.422	0.005	0.016	0.003	-0.002	-0.628
N42	N80	3.586	0.004	0.014	0.003	-0.002	-0.628
N45	N67	3.464	0.005	0.019	0.004	-0.003	-0.941
N45	N88	3.586	0.004	0.013	0.002	-0.002	-0.628
N48	N84	4.692	0.0005	0.0014	0.0002	-0.0001	-0.031

Atom 1	Atom 2	Distance	$\rho(r_{cp})$	$\nabla^2\rho(r_{cp})$	$G(r_{cp})$	$V(r_{cp})$	E_{int}
**^{ED}[MTX-1*·Rb(H_2O)]$_n$**							
N1	N4	3.457	0.039	0.003	0.003	-0.002	-0.678
N1	C1	3.454	0.037	0.004	0.003	-0.002	-0.712
N3	C1	3.601	0.039	0.003	0.003	-0.002	-0.649
N4	N1	3.457	0.039	0.003	0.003	-0.002	-0.675
C1	N3	3.601	0.039	0.003	0.003	-0.002	-0.646
N5	N2	3.422	0.031	0.003	0.003	-0.002	-0.596
N8	N8	3.464	0.038	0.004	0.004	-0.003	-0.803
N8	N8	3.587	0.028	0.003	0.002	-0.002	-0.533
**[MTX-1*·Cs(H_2O)]$_n$**							
N2	N47	3.767	0.003	0.008	0.002	-0.001	-0.314
N3	N22	3.296	0.005	0.019	0.004	-0.003	-0.941
N3	N45	3.516	0.004	0.013	0.002	-0.002	-0.628
N4	C53	3.383	0.005	0.016	0.003	-0.003	-0.941
C13	N21	3.383	0.005	0.016	0.003	-0.003	-0.941
N5	N42	3.296	0.005	0.019	0.004	-0.003	-0.941
N6	N20	3.516	0.004	0.013	0.002	-0.002	-0.628
N19	N7	3.767	0.003	0.008	0.002	-0.001	-0.314
**(MTX-1*)$_2$·Mg(H_2O)$_6$**							
N22	N73	3.443	0.004	0.015	0.003	-0.002	-0.628
N22	C36	3.168	0.007	0.026	0.005	-0.004	-1.255
C20	C71	3.390	0.005	0.016	0.003	-0.002	-0.628
N26	C65	3.390	0.005	0.016	0.003	-0.003	-0.941
N26	N42	3.311	0.005	0.020	0.004	-0.003	-0.941
N27	N41	3.311	0.005	0.020	0.004	-0.003	-0.941
N28	N67	3.443	0.004	0.015	0.003	-0.002	-0.628
N28	C35	3.334	0.006	0.020	0.004	-0.003	-0.941
C21	N59	3.293	0.006	0.020	0.004	-0.003	-0.941
N32	N37	3.276	0.007	0.026	0.005	-0.004	-1.255
N29	C51	3.293	0.006	0.020	0.004	-0.003	-0.941

Atom 1	Atom 2	Distance	$\rho(\mathbf{r}_{cp})$	$\nabla^2\rho(\mathbf{r}_{cp})$	$G(\mathbf{r}_{cp})$	$V(\mathbf{r}_{cp})$	E_{int}
**[(MTX-1*)$_2$·Ca(H$_2$O)$_4$]**							
N8	N29	3.568	0.005	0.016	0.003	-0.002	-0.628
N8	C171	3.234	0.007	0.022	0.005	-0.004	-1.255
N10	N104	3.558	0.004	0.013	0.003	-0.002	-0.628
N11	N61	3.348	0.006	0.021	0.004	-0.003	-0.941
C21	N101	3.200	0.007	0.025	0.005	-0.004	-1.255
C21	N158	3.234	0.007	0.022	0.005	-0.004	-1.255
N12	N162	3.327	0.005	0.017	0.003	-0.002	-0.628
N13	N58	3.205	0.006	0.023	0.005	-0.004	-1.255
N13	N99	3.347	0.004	0.016	0.003	-0.002	-0.628
N15	N56	3.205	0.006	0.023	0.005	-0.004	-1.255
N15	C107	3.200	0.007	0.025	0.005	-0.004	-1.255
N18	N54	3.348	0.006	0.013	0.004	-0.003	-0.941
N18	N96	3.910	0.004	0.021	0.003	-0.002	-0.628
**(MTX-1*)·[MTX-1*·Sr(H$_2$O)$_6$]$_n$**							
N33	N80	3.337	0.006	0.020	0.004	-0.003	-0.941
N34	N81	3.326	0.006	0.020	0.004	-0.003	-0.941
N35	N67	3.355	0.005	0.020	0.004	-0.003	-0.941
N33	N66	3.317	0.006	0.021	0.004	-0.003	-0.941
N35	C92	3.345	0.005	0.018	0.004	-0.003	-0.941
N36	C77	3.253	0.006	0.021	0.004	-0.003	-0.941
N36	N79	3.382	0.005	0.018	0.004	-0.002	-0.628
C46	N65	3.327	0.005	0.019	0.004	-0.003	-0.941
N38	N104	3.276	0.006	0.020	0.004	-0.003	-0.941
N39	N13	3.418	0.005	0.017	0.003	-0.002	-0.628
N40	N10	3.459	0.005	0.017	0.003	-0.002	-0.628
N40	N101	3.435	0.005	0.018	0.003	-0.002	-0.628
N43	N9	3.338	0.006	0.021	0.004	-0.003	-0.941
**(MTX-1*)·[MTX-1*·Ba(H$_2$O)$_6$]$_n$**							
N16	N76	3.350	0.006	0.020	0.004	-0.003	-0.941
N17	N46	3.339	0.006	0.020	0.004	-0.003	-0.941
N16	N45	3.333	0.006	0.020	0.004	-0.003	-0.941
N18	C57	3.340	0.005	0.018	0.004	-0.003	-0.941
N18	N77	3.385	0.005	0.018	0.004	-0.003	-0.941
N19	N44	3.406	0.005	0.018	0.003	-0.002	-0.628

N19	C87	3.287	0.006	0.019	0.004	-0.003	-0.941
Atom 1	Atom 2	Distance	$\rho(r_{cp})$	$\nabla^2\rho(r_{cp})$	$G(r_{cp})$	$V(r_{cp})$	E_{int}
C29	N75	3.346	0.005	0.019	0.004	-0.003	-0.941
N20	N75	3.355	0.005	0.019	0.004	-0.003	-0.941
N21	N66	3.307	0.005	0.019	0.004	-0.003	-0.941
N22	N96	3.430	0.005	0.017	0.003	-0.002	-0.628
N23	N65	3.562	0.004	0.015	0.003	-0.002	-0.628
N23	N95	3.365	0.006	0.020	0.004	-0.003	-0.941
N26	N69	3.435	0.005	0.017	0.003	-0.002	-0.628
N26	N99	3.492	0.005	0.016	0.003	-0.002	-0.628

*$\rho(r_{cp})$ – the electron density, $\nabla^2\rho(r_{cp})$ – the Laplacian function of the electron density, $G(r_{cp})$ – the kinetic-electron energy density, $V(r_{cp})$ – the potential-electron energy density.

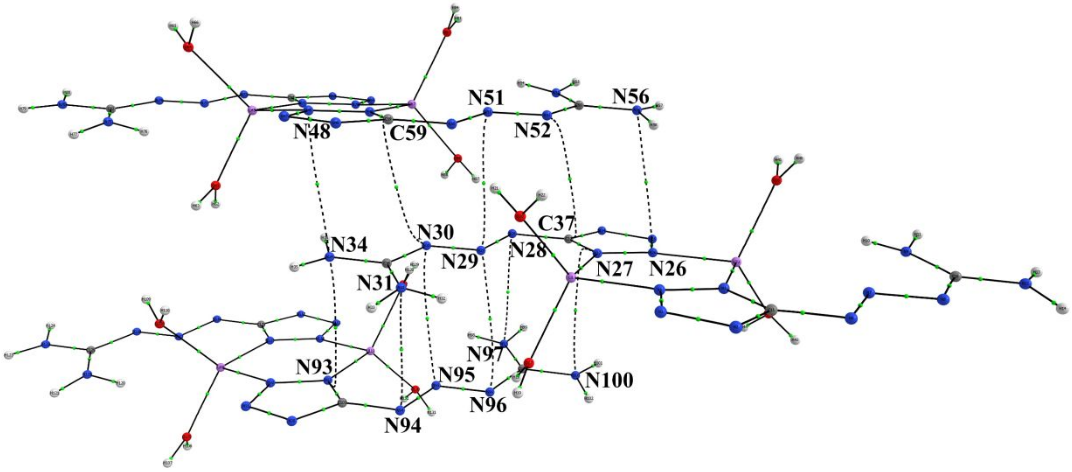

Figure S26. The molecular graph for key fragment of unit cell of **[MTX-1*·Li(H_2O)$_2$]$_2$**. Only BCPs (3, -1) are presented for clarity (green). The dashed line marks the bonding pathways involved in π-π stacking for one molecule of **MTX-1***, the others are omitted for clarity.

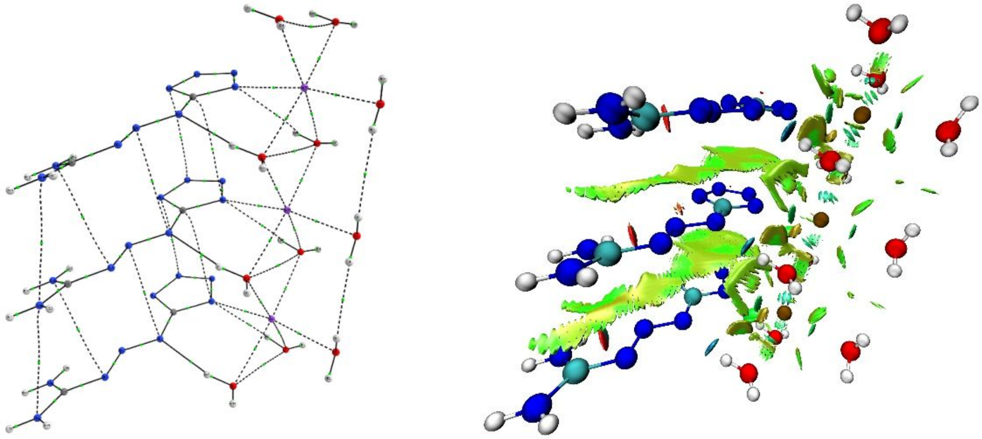

Figure S27. The molecular graph and NCI plot of gradient isosurfaces (s=0.5 au) for key fragment of unit cell of **[MTX-1*·Na(H_2O)$_3$]$_n$**. Only BCPs (3, -1) are presented for clarity (green). The surfaces are colored on a blue-green-red (BGR) scale, ranging from -0.04 to 0.02 au.

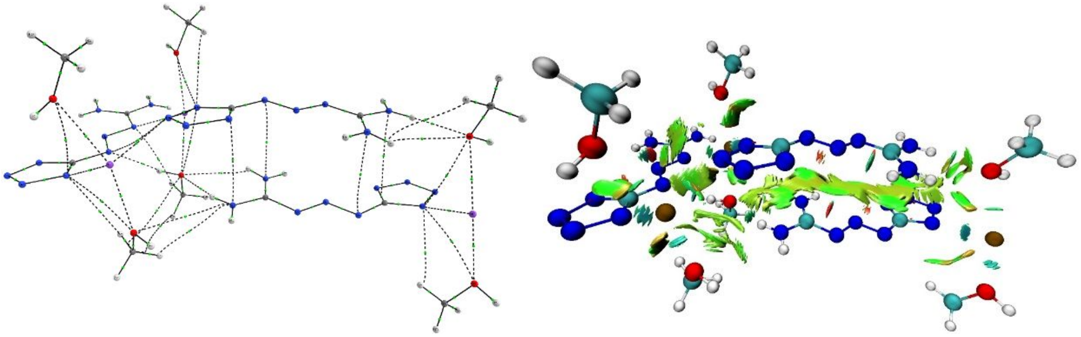

Figure S28. The molecular graph and NCI plot of gradient isosurfaces (s=0.5 au) for key fragment of unit cell of **[MTX-1*·Na(MeOH)$_2$]$_n$**. Only BCPs (3, -1) are presented for clarity (green). The surfaces are colored on a blue-green-red (BGR) scale, ranging from -0.04 to 0.02 au.

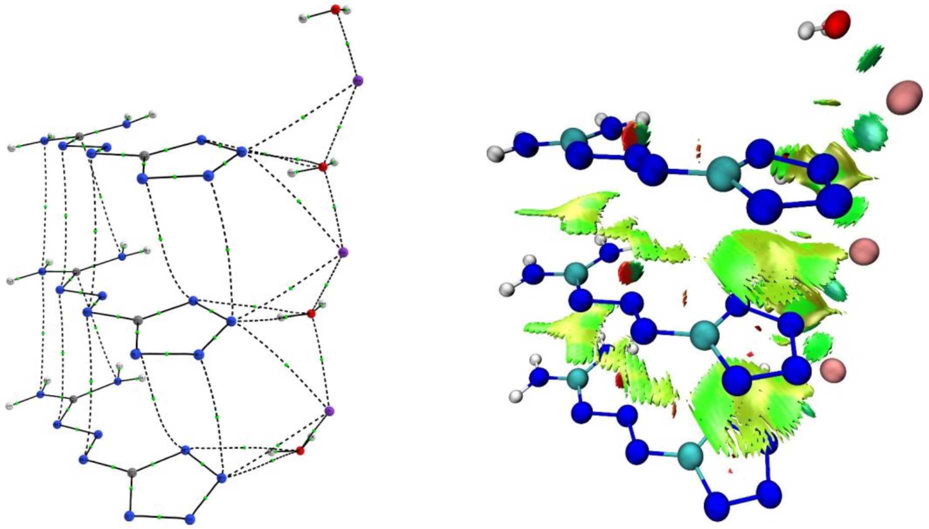

Figure S29. The molecular graph and NCI plot of gradient isosurfaces (s=0.5 au) for key fragment of unit cell of **[MTX-1*·K(H$_2$O)]$_n$**. Only BCPs (3, -1) are presented for clarity (green). The surfaces are colored on a blue-green-red (BGR) scale, ranging from -0.04 to 0.02 au.

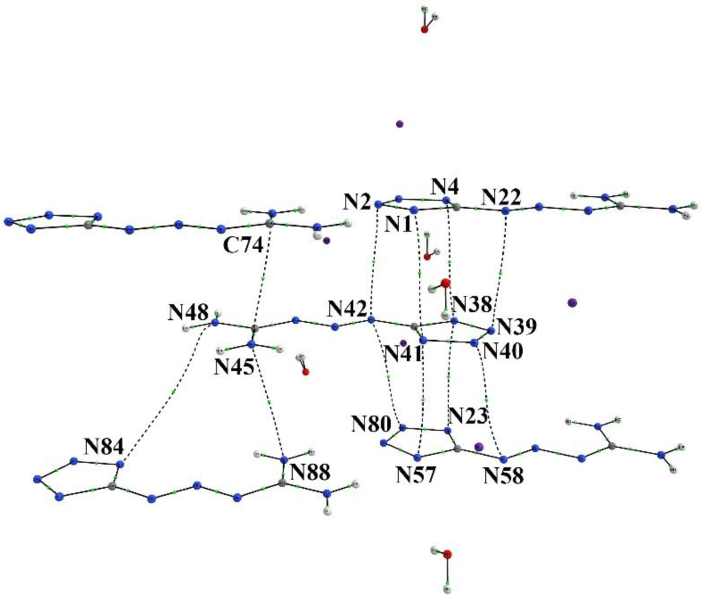

Figure S30. The molecular graph for key fragment of unit cell of **[MTX-1*·Rb(H$_2$O)]$_n$**. Only BCPs (3, -1) are presented for clarity (green). The dashed line marks the bonding pathways involved in π-π stacking for one molecule of **MTX-1***, the others are omitted for clarity.

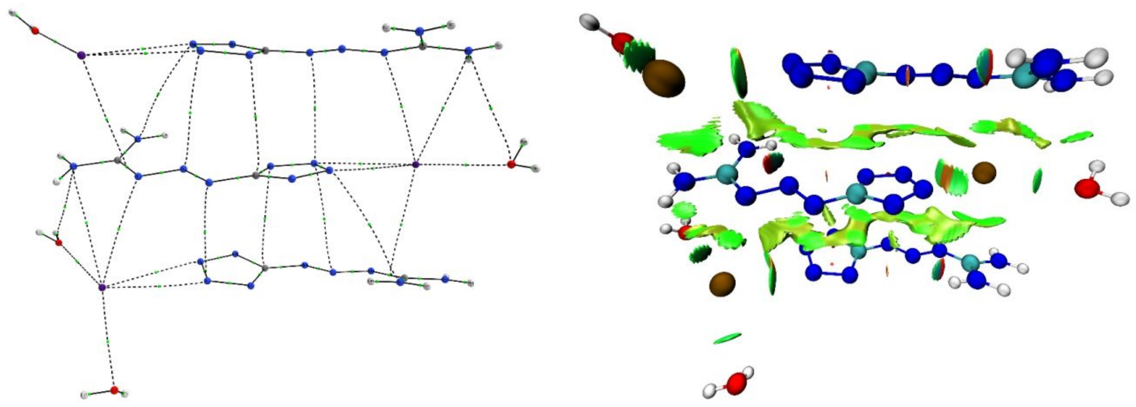

Figure S31. The molecular graph and NCI plot of gradient isosurfaces (s=0.5 au) for key fragment of unit cell of **[MTX-1*·Cs(H_2O)]$_n$**. Only BCPs (3, -1) are presented for clarity (green). The surfaces are colored on a blue-green-red (BGR) scale, ranging from -0.04 to 0.02 au.

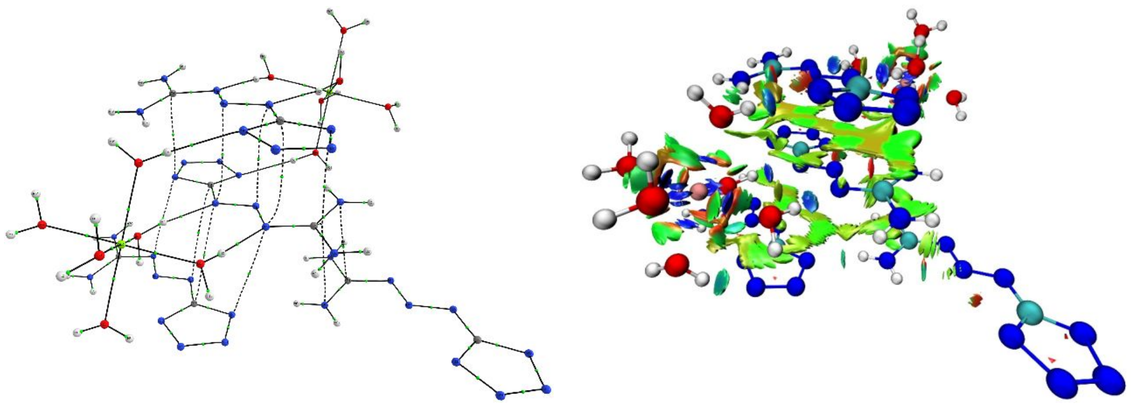

Figure S32. The molecular graph and NCI plot of gradient isosurfaces (s=0.5 au) for key fragment of unit cell of **(MTX-1*)$_2$·Mg(H_2O)$_6$**. Only BCPs (3, -1) are presented for clarity (green). The dashed line marks the bonding pathways involved in π-π stacking for one molecule of **MTX-1***, the others are omitted for clarity. The surfaces are colored on a blue-green-red (BGR) scale, ranging from -0.04 to 0.02 au.

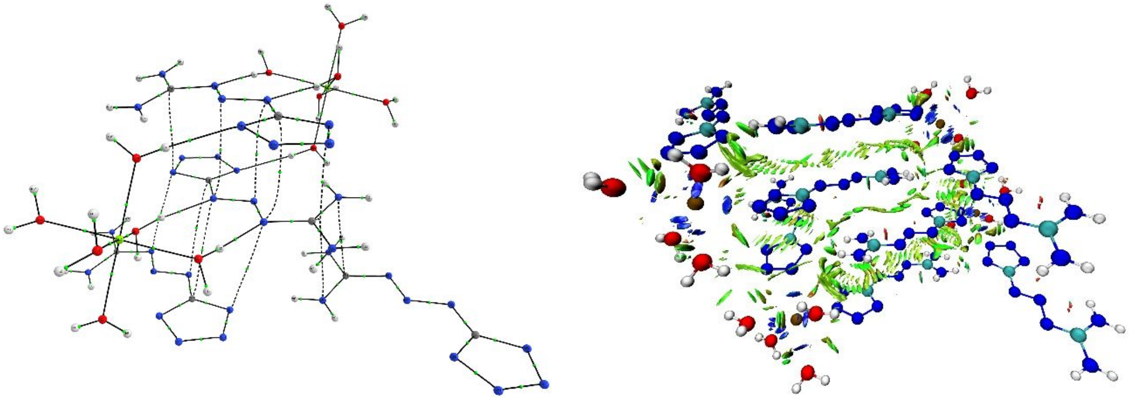

Figure S33. The molecular graph and NCI plot of gradient isosurfaces (s=0.5 au) for key fragment of unit cell of **[(MTX-1*)$_2$·Ca(H$_2$O)$_4$]**. Only BCPs (3, -1) are presented for clarity (green). The dashed line marks the bonding pathways involved in π-π stacking for one molecule of **MTX-1***, the others are omitted for clarity. The surfaces are colored on a blue-green-red (BGR) scale, ranging from -0.04 to 0.02 au.

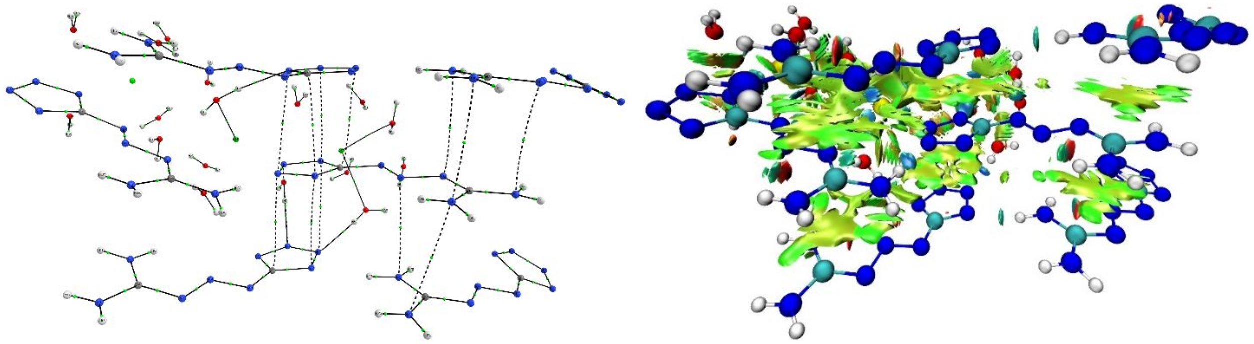

Figure S34. The molecular graph and NCI plot of gradient isosurfaces (s=0.5 au) for key fragment of unit cell of **(MTX-1*)·[MTX-1*·Sr(H$_2$O)$_6$]$_n$**. Only BCPs (3, -1) are presented for clarity (green). The dashed line marks the bonding pathways involved in π-π stacking for one molecule of **MTX-1***, the others are omitted for clarity. The surfaces are colored on a blue-green-red (BGR) scale, ranging from -0.04 to 0.02 au.

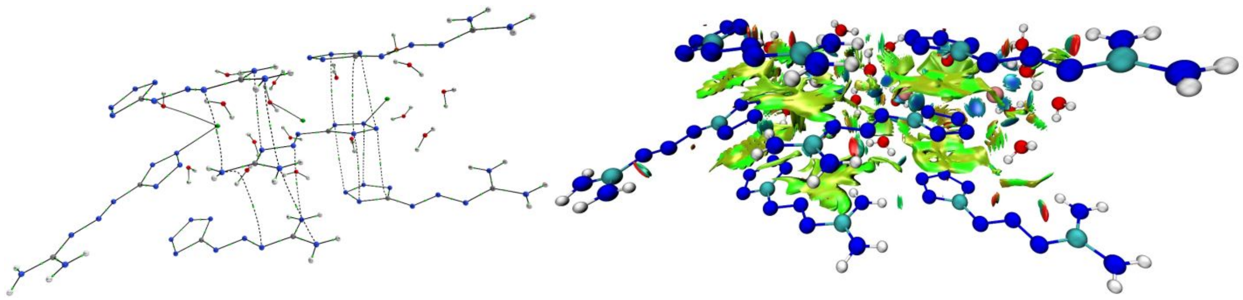

Figure S35. The molecular graph and NCI plot of gradient isosurfaces (s=0.5 au) for key fragment of unit cell of **(MTX-1*)·[MTX-1*·Ba(H$_2$O)$_6$]$_n$**. Only BCPs (3, −1) are presented for clarity (green). The dashed line marks the bonding pathways involved in π-π stacking for one molecule of **MTX-1***, the others are omitted for clarity. The surfaces are colored on a blue-green-red (BGR) scale, ranging from −0.04 to 0.02 au.

References:

(1) Šelešovský, J.; Pachman, J., Probit Analysis – a Promising Tool for Evaluation of Explosive's Sensitivity *). *Central European Journal of Energetic Materials* **2010,** *7.*

(2) Musil, T.; Matyáš, R.; Vala, R.; Růžička, A.; Vlček, M., Silver Salt of 4,6-Diazido-N-nitro-1,3,5-triazine-2-amine – Characterization of this Primary Explosive. *Propellants, Explosives, Pyrotechnics* **2014,** *39* (2), 251-259.

(3) *Recommendations on the Transport of Dangerous Goods, Manual of Tests and Criteria*. 7th rev. ed. ed.; United Nations: New York, 2019.

(4) Bruker-AXS *APEX4 v2022.1-1*, 2022.

(5) Hansen, N. K.; Coppens, P., Testing aspherical atom refinements on small-molecule data sets. *Acta Crystallographica Section A* **1978,** *34* (6), 909-921.

(6) Jelsch, C.; Guillot, B.; Lagoutte, A.; Lecomte, C., Advances in protein and small-molecule charge-density refinement methods using MoPro. *Journal of Applied Crystallography* **2005,** *38* (1), 38-54.

(7) Allen, F. H.; Bruno, I. J., Bond lengths in organic and metal-organic compounds revisited: X-H bond lengths from neutron diffraction data. *Acta Crystallographica Section B* **2010,** *66* (3), 380-386.

(8) Hirshfeld, F., Can X-ray data distinguish bonding effects from vibrational smearing? *Acta Crystallographica Section A* **1976,** *32* (2), 239-244.

(9) Guillot, B., MoProViewer: a molecule viewer for the MoPro charge-density analysis program. *Acta Crystallographica Section A* **2012,** *68* (a1), s204.

(10) Frisch, M. J.; Trucks, G. W.; Schlegel, H. B.; Scuseria, G. E.; Robb, M. A.; Cheeseman, J. R.; Scalmani, G.; Barone, V.; Petersson, G. A.; Nakatsuji, H.; Li, X.; Caricato, M.; Marenich, A. V.; Bloino, J.; Janesko, B. G.; Gomperts, R.; Mennucci, B.; Hratchian, H. P.; Ortiz, J. V.; Izmaylov, A. F.; Sonnenberg, J. L.; Williams; Ding, F.; Lipparini, F.; Egidi, F.; Goings, J.; Peng, B.; Petrone, A.; Henderson, T.; Ranasinghe, D.; Zakrzewski, V. G.; Gao, J.; Rega, N.; Zheng, G.; Liang, W.; Hada, M.; Ehara, M.; Toyota, K.; Fukuda, R.; Hasegawa, J.; Ishida, M.; Nakajima, T.; Honda, Y.; Kitao, O.; Nakai, H.; Vreven, T.; Throssell, K.; Montgomery Jr., J. A.; Peralta, J. E.; Ogliaro, F.; Bearpark, M. J.; Heyd, J. J.; Brothers, E. N.; Kudin, K. N.; Staroverov, V. N.; Keith, T. A.; Kobayashi, R.; Normand, J.; Raghavachari, K.; Rendell, A. P.; Burant, J. C.; Iyengar, S. S.; Tomasi, J.; Cossi, M.; Millam, J. M.; Klene, M.; Adamo, C.; Cammi, R.; Ochterski, J. W.; Martin, R. L.; Morokuma, K.; Farkas, O.; Foresman, J. B.; Fox, D. J. *Gaussian 16 Rev. C.01*, Wallingford, CT, 2016.

(11) Hellweg, A.; Rappoport, D., Development of new auxiliary basis functions of the Karlsruhe segmented contracted basis sets including diffuse basis functions (def2-SVPD, def2-TZVPPD, and def2-QVPPD) for RI-MP2 and RI-CC calculations. *Physical Chemistry Chemical Physics* **2015,** *17* (2), 1010-1017.

(12) Grimme, S.; Antony, J.; Ehrlich, S.; Krieg, H., A consistent and accurate ab initio parametrization of density functional dispersion correction (DFT-D) for the 94 elements H-Pu. *The Journal of Chemical Physics* **2010,** *132* (15), 154104.

(13) Zhao, Y.; Truhlar, D. G., The M06 suite of density functionals for main group thermochemistry, thermochemical kinetics, noncovalent interactions, excited states, and transition elements: two new functionals and systematic testing of four M06-class functionals and 12 other functionals. *Theoretical Chemistry Accounts* **2008,** *120* (1), 215-241.

(14) Johnson, E. R.; Keinan, S.; Mori-Sánchez, P.; Contreras-García, J.; Cohen, A. J.; Yang, W., Revealing Noncovalent Interactions. *Journal of the American Chemical Society* **2010,** *132* (18), 6498-6506.
(15) Lu, T., A comprehensive electron wavefunction analysis toolbox for chemists, Multiwfn. *The Journal of Chemical Physics* **2024,** *161* (8).
(16) Humphrey, W.; Dalke, A.; Schulten, K., VMD: Visual molecular dynamics. *Journal of Molecular Graphics* **1996,** *14* (1), 33-38.
(17) AIMAll (Version 19.10.12), T. A. K., TK Gristmill Software, Overland Park KS, USA, 2019 (aim.tkgristmill.com).
(18) Ryšavý, J.; Matyáš, R., Effects of crystal habit modifiers on characterization parameters of tetrazene. *Scientific Papers of the University of Pardubice* **2021**, 227-270.
(19) Fronabarger, J. W.; Williams, M. D.; Stern, A. G.; Parrish, D. A., MTX-1 – A Potential Replacement for Tetrazene in Primers. *Central European Journal of Energetic Materials* **2016,** *13*, 33-52.
(20) Fronabarger, J. W.; Williams, M. D. Alternative to tetrazene. US8524019, 2013.
